# Supramolecular Phase Change Materials for Spatiotemporally Thermal Energy Utilization

**DOI:** 10.1002/advs.202512924

**Published:** 2025-12-12

**Authors:** Miaomiao Yan, Cong Liu, Ruolan Tang, Xueyu Zhu, Zongbin Li, Jiong Zhou

**Affiliations:** ^1^ Department of Chemistry College of Sciences Northeastern University Shenyang 110819 China; ^2^ Key Laboratory for Anisotropy and Texture of Materials (Ministry of Education) School of Materials Science and Engineering Northeastern University Shenyang 110819 China

**Keywords:** controllable release, phase change materials, pillararenes, supramolecular chemistry, thermal energy storage

## Abstract

Phase change materials (PCMs) are promising heat storage media to solve the intermittency and instability of renewable energy utilization. However, due to the spontaneous crystallization behavior and the accompanied release of latent heat upon cooling, the absorbed thermal energy can not be well stored at room temperature, which severely limits the applicability of PCMs in thermal energy storage. Herein, the long‐term storage as well as switchable and controllable release of thermal energy using activated perethylated pillar[5]arene **EtP5** (**EtP5**α) is reported for the first time. Through activation at 393 K, **EtP5**α can store thermal energy in the supercooled state at room temperature and release thermal energy by triggering cold crystallization at 370 K. High thermal energy storage capacity can be maintained for 20 thermal cycles and more than 365 days at room temperature, which is the PCMs that can store thermal energy for the longest time at room temperature.

## Graphical abstract

The long‐term storage as well as switchable and controllable release of thermal energy using activated perethylated pillar[5]arene **EtP5** (**EtP5**α) is demonstrated for the first time. High thermal energy storage capacity can be maintained over 20 thermal cycles and for more than 365 days at room temperature, which is the PCMs that can store thermal energy for the longest time at room temperature.

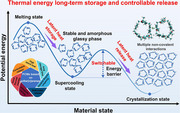

## 1 Introduction

With the increasing demand for energy in human production and life, the exploitation and utilization of environment friendly sustainable energy, such as solar energy, wind energy, geothermal energy, and tidal energy, have become a research hotspot.^[^ ^1^, ^2^, ^3^ ^]^ However, the use of renewable energy in some key areas is restricted due to the intermittency and instability.^[^ ^4^, ^5^ ^]^ Thus, developing efficient and long‐term stable energy storage technologies is of great importance for the renewable energy utilization.^[^ ^6^, ^7^, ^8^, ^9^, ^10^ ^]^ Thermal energy storage, including sensible heat, latent heat and thermochemical heat storage, has been well explored as a promising energy storage technology to solve the dilemma between the energy supply and demand in time and space.^[^ ^11^, ^12^, ^13^, ^14^, ^15^ ^]^ Among them, owing to the high energy storage capacity, small temperature fluctuation and outstanding thermal stability, latent heat storage shows good potential for thermal energy storage application.^[^ ^16^, ^17^ ^]^

Phase change materials (PCMs) are considered to be ideal latent heat storage media for their special ability to absorb and release large amounts of latent heat during the phase change process.^[^ ^18^, ^19^, ^20^, ^21^, ^22^, ^23^ ^]^ Various types of PCMs, such as inorganic salts, organic polyols, and aliphatic compounds, are used for heat storage over different operating temperature ranges.^[^ ^24^, ^25^, ^26^ ^]^ Although these PCMs can absorb considerable thermal energy upon the heating process, the absorbed thermal energy will be spontaneously released during the cooling process due to the cold crystallization. Thereby, a high storage temperature is required to avoid the possible spontaneous energy release, which significantly limits the applicability of thermal energy storage in most fields.^[^ ^27^, ^28^, ^29^, ^30^ ^]^ Additionally, long‐term thermal energy storage can further extend the storage period and eliminate the intermittency issues of renewable energy technologies, allowing thermal energy systems to operate stably regardless of weather conditions and transportation distances.^[^ ^31^, ^32^ ^]^ But more than 30% of the latent heat stored by these PCMs is gradually lost to the environment over several months even with sophisticated insulation.^[^ ^33^ ^]^ Such uncontrollable thermal energy release and short energy storage time are the main obstacles to the thermal energy storage and utilization of traditional PCMs.^[^ ^34^ ^]^

Previous studies have introduced the controlled thermal energy release of some photo‐switching molecules (such as azobenzene) into organic PCMs.^[^ ^35^, ^36^, ^37^, ^38^ ^]^ Owing to the intermolecular interaction between photo‐switching molecules and PCMs, the spontaneous crystallization is suppressed.^[^ ^39^ ^]^ As a result, the organic PCMs are in supercooled state, and most of the absorbed thermal energy can be stored below the crystallization temperature.^[^ ^40^ ^]^ However, it is difficult to apply photo‐switching molecules to the thermal energy long‐term storage because of their metastable nature.^[^ ^41^ ^]^ In addition, the manufacturing of such PCM composites often needs complex synthesis processes and long periods of UV light for isomerization.^[^ ^42^ ^]^ Therefore, it is urgent to explore novel PCMs with simple synthesis, long‐term thermal energy storage and controllable release of thermal energy.^[^ ^43^, ^44^ ^]^

In recent years, supramolecular chemistry has attracted widespread attention in energy‐related fields on account of its intelligent responsiveness to external stimuli, as well as its reversibility and reusability.^[^ ^45^, ^46^, ^47^ ^]^ Supramolecular chemistry is “chemistry beyond molecules”.^[^ ^48^, ^49^, ^50^, ^51^ ^]^ In particular, pillar[*n*]arenes, as a new generation of macrocycles, have rigid structures and unique pillar‐shapes through connecting hydroquinone units with methylene bridges at *para*‐position.^[^ ^52^, ^53^, ^54^ ^]^ With the advantages of simple preparation, good chemical and thermal stability, pillar[*n*]arenes have been widely used in many fields, such as adsorptive separation, ion recognition and biomedicine.^[^ ^55^, ^56^, ^57^, ^58^, ^59^ ^]^ However, to the best of our knowledge, the application of pillar[*n*]arenes in thermal energy storage has not been studied. Some recent works have demonstrated that pillar[*n*]arenes‐based polymers exhibit strong structural reversibility due to non‐covalent interactions.^[^ ^60^, ^61^, ^62^ ^]^ Additionally, reversible thermally induced phase transitions have been observed in pillar[*n*]arenes‐based liquid crystals prepared by bottom‐up synthesis of small molecular components through non‐covalent cross‐linked networks.^[^ ^63^ ^]^ These studies indicate that the dynamic and reversible non‐covalent interactions endow pillar[*n*]arenes with reversible structural phase transitions, which may hold potential in the field of phase change thermal energy storage.

Herein, we demonstrate controllable thermal energy release and long‐term thermal energy storage in the activated **EtP5** (**EtP5** *α*). Due to multiple non‐covalent interactions between **EtP5** *α* molecules, **EtP5** *α* exhibit reversible phase transition during the thermal cycle, yielding stable heat release and absorption. Besides, the large energy barrier postpones the crystallization behavior and the corresponding heat release. Consequently, the absorbed heat can be stored at room temperature for more than 365 days. Moreover, through introducing expanded graphite to manipulate the energy barrier, thermal energy release can be switched into the desired temperature region. With the combination of high latent heat, controllable and switchable thermal energy release, long heat storage time, excellent cycling stability and thermal stability, **EtP5** *α* become extremely attractive in the field of thermal energy storage. This is the first example of long‐term storage as well as controllable and switchable release of thermal energy discovered in the field of supramolecular macrocycles, which will provide a promising direction for the research on thermal energy storage and release.

## 2 Results and Discussion

### 2.1 Preparation and Characterization of EtP5*α*

**EtP5** *α* were synthesized and activated by the previously reported method.^[^ ^64^ ^] 1^H NMR (Figure S1, Supporting Information) and thermogravimetric analysis (TGA, Figure S2, Supporting Information) verified that solvents were removed. Power X‐ray diffraction (PXRD, Figure S3, Supporting Information) demonstrated that **EtP5** *α* were crystalline.

### 2.2 Phase Change Behaviors

**Figure 1:**
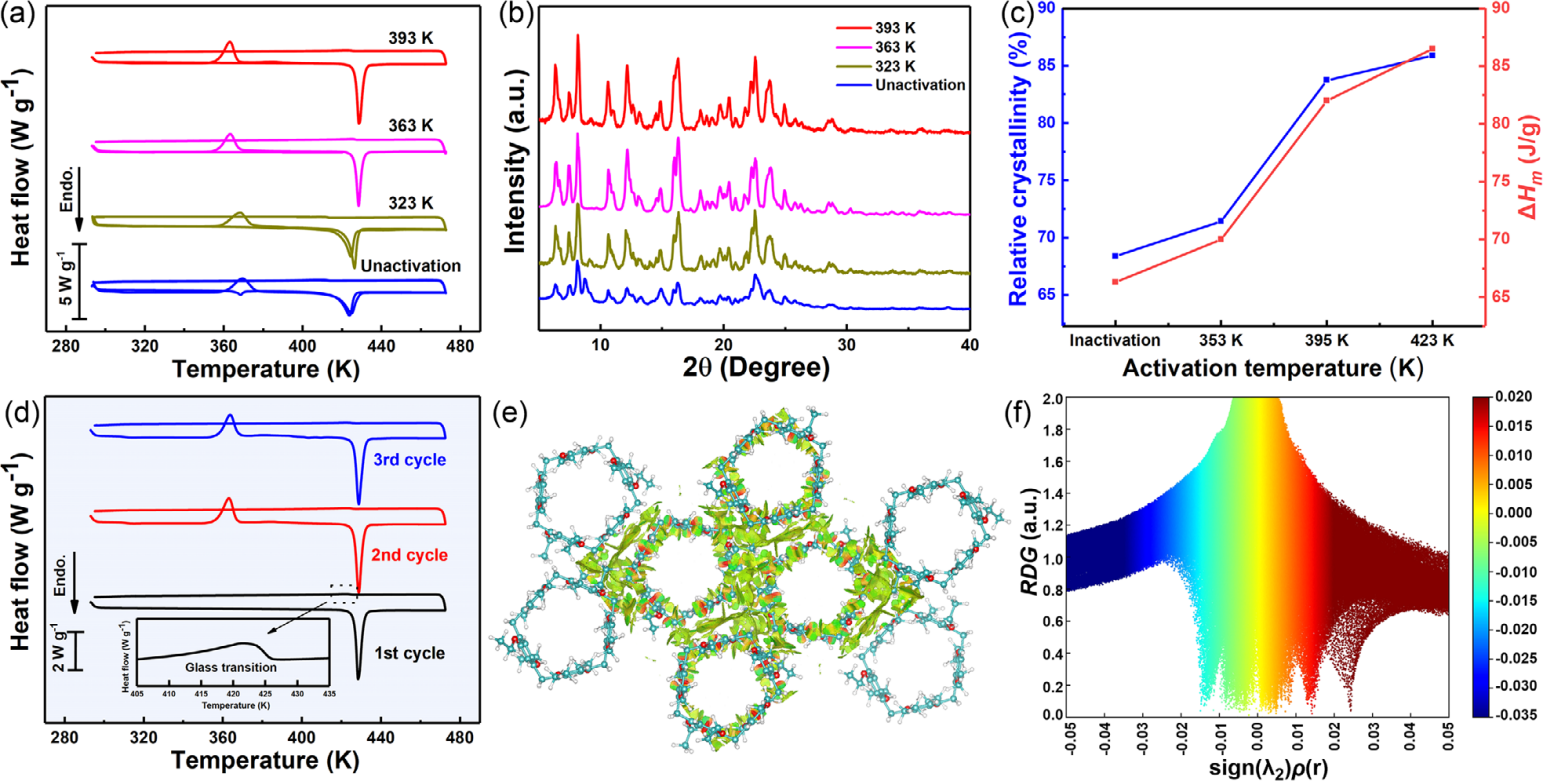
a) DSC curves of unactivated **EtP5** and **EtP5** *α* at different activation temperatures. b) PXRD patterns of **EtP5** *α* at different activation temperatures. c) Relative crystallinity and melting enthalpy (Δ*H_m_*) of **EtP5** *α* at different activation temperatures. d) DSC curves of **EtP5** *α* activated at 393 K for 24 h. Non‐covalent interaction scatter diagram e) and reduced density gradient analysis (f) of **EtP5** *α*.

Phase change behaviors of **EtP5** *α* were characterized by differential scanning calorimetry (DSC). The DSC measurement showed that unactivated **EtP5** exhibited two downward endothermic peaks during the first heating process (**Figure** **1**a). The first endothermic peak at ≈368 K referred to the solid‐solid phase transition, while the second endothermic peak at ≈423 K was associated with the melting. Upon the cooling process, only a slight inflection point was observed at ≈421 K, indicating that the sample only underwent glass transition rather than crystallization, and was in the supercooled state at room temperature. Notably, the first endothermic peak vanished and an exothermic peak appeared at 368 K during the second heating process. Besides, in the cases of **EtP5** activated at 323–393 K for 24 h, no endothermic peak at 368 K was detected during the first heating process. Thus, it could be concluded that the endothermic behavior of the unactivated **EtP5** at 368 K was caused by the phase transformation from **EtP5** with low‐crystallinity to **EtP5** *α* with high‐crystallinity. The PXRD results of the unactivated **EtP5** and **EtP5** *α* before the thermal cycles also confirmed that the high activation temperature could improve the crystallinity (Figure 1b). In addition, the melting enthalpy (Δ*H_m_*) also increased with the enhanced activation temperature, which may be the result of the improved crystallinity (Figure 1c).

It is well known that the thermal energy storage capacity is highly dependent on the latent heat absorbed and released during the phase transformation.^[^ ^65^ ^]^ Hereafter, the focus was paid on the thermal energy storage behavior of **EtP5** activated at 393 K because of the relatively high Δ*H_m_*. The DSC curves showed that a considerable thermal energy of 86.5 J g^−1^ was absorbed during the melting process and well stored during the cooling process (Figure 1d). It was not until the supercooled phase was reheated to above 363 K that the cold crystallization of the supercooled phase was triggered, thereby releasing the stored latent heat. Such thermal energy storage performance was temperature‐controlled and reversible, which conferred **EtP5** *α* huge potential to be implemented in the thermal energy storage applications. Additionally, reheating is not the only method to induce cold crystallization. Alternative triggers, such as ultrasound, mechanical stirring, microwave or adding seeds can also be effective.^[^ ^66^, ^67^, ^68^, ^69^ ^]^ **EtP5** *α* were transferred to ultrasonic device for triggering tests, and the results showed that ultrasonication could achieve the exothermic temperature of supercooled phase (Figure S4, Supporting Information). In addition, ultrasonication reduced the phase transition temperature of **EtP5** *α* (Figure S5, Supporting Information). In contrast, the monomer of **EtP5** *α*, *p*‐diethoxybenzene, began to melt at 345 K upon heating while spontaneously crystallized at 325 K upon cooling (Figure S6, Supporting Information). During the second thermal cycle, no endothermic and exothermic peaks were observed, demonstrating that the thermal energy storage behavior of *p*‐diethoxybenzene was irreversible. In addition, the single crystal structure revealed that there were C–H···O and C–H···*π* interactions between **EtP5** *α* molecules (C−H···*π* distance: 2.885 Å; C−H···O distances: 2.656 Å, 2.840 Å) (Figures S7 and S8, Supporting Information). The multiple non‐covalent interactions could prevent them from spontaneous crystallization during the cooling process, which might be the reason for the controllable and reversible thermal energy storage behavior of **EtP5** *α* (Figure 1e,f). This was the first time that the controllable and reversible storage and release of thermal energy observed in the field of supramolecular macrocycles

### 2.3 Structural Analysis of EtP5*α* During Heating and Cooling Cycles

**Figure 2:**
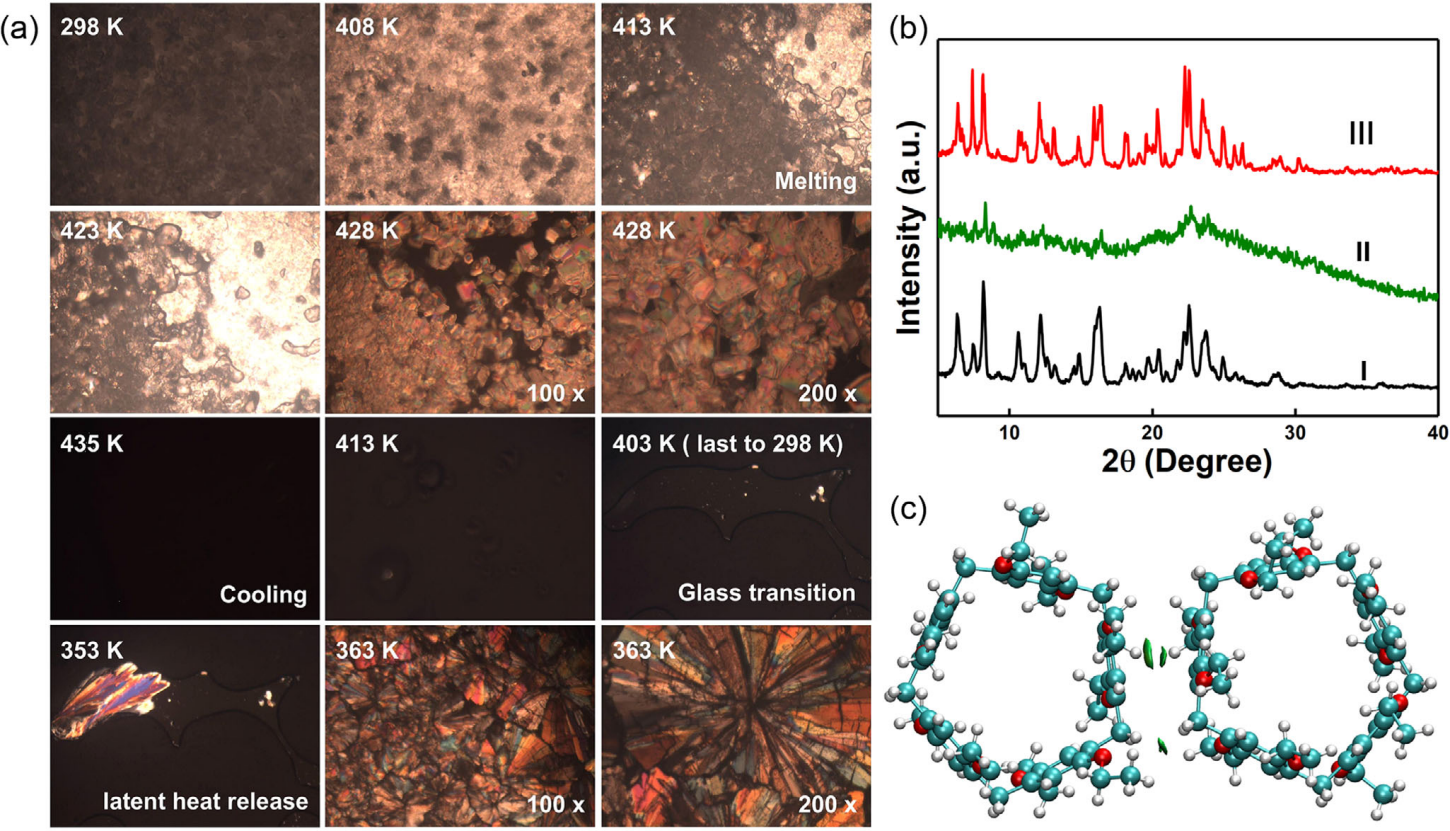
a) Optical micrographs of **EtP5** *α* during the thermal energy storage and controllable release. b) PXRD patterns of: I) original **EtP5** *α*; II) **EtP5** *α* after glass transition; III) reheated **EtP5** *α* after glass transition. c) Multiple non‐covalent interactions between **EtP5** *α* molecules.

The melting and crystallization behaviors of **EtP5** *α* during thermal cycles were directly observed by optical microscopy (**Figure** **2**a). Optical micrographs showed that **EtP5** *α* were in solid state at temperatures ranging from 298 K to 408 K. As the temperature exceeded above 413 K, **EtP5** *α* began to melt, featured by the gradual disappearance of regular and colorful blocks. When heated to 435 K, **EtP5** *α* completely melted and the colored crystals quickly disappeared. Then **EtP5** *α* underwent glass transition into non‐refractive state as the temperature decreased. The amorphous structure did not undergo crystalline transition and existed in the transparent glassy state. When reheated to 353 K, the glassy phase gradually crystallized into colored flake crystals.

The PXRD results showed the structural changes of **EtP5** *α* at different states (Figure 2b). The PXRD pattern of the supercooled phase after the glass transition showed a broad bulge, which was a typical indication of the amorphous phase. However, as the supercooled phase was reheated to 363 K, the amorphous structure was converted back to **EtP5** *α*. This change in crystal structure might be the result of the dynamic and reversible non‐covalent interactions between **EtP5** *α* molecules (Figure 2c), which guaranteed the cyclicity of thermal energy storage and release. In situ infrared spectroscopy showed that the stretching vibration peaks of the C–H bonds were blue‐shifted toward higher wavenumbers upon **EtP5** *α* were heated (Figure S9, Supporting Information). When reheating supercooled phase, the heat energy was released. The non‐covalent interactions between **EtP5** *α* molecules were restored, accompanied by a redshift in the C–H stretching vibration. The above results revealed the cold crystallization transition from the amorphous glass phase to the high crystallinity **EtP5** *α*.

Additionally, the morphological characteristics of **EtP5** *α* before and after thermal cycling were observed using scanning electron microscopy (SEM) and high resolution transmission electron microscopy (HR‐TEM). **EtP5** *α* displayed regular dispersed block morphology (Figure S10a, Supporting Information). By HR‐TEM analysis, the crystalline interplanar spacing were 0.216 and 0.228 nm, respectively (Figure S10b–d, Supporting Information). After the glass transition, the supercooled particles clustered together into agglomerates with irregular stripes (Figure S10e–g, Supporting Information). The diffusive ring without diffraction spots further confirmed the amorphous nature of the supercooled phase (Figure S10h, Supporting Information). Upon reheating, the HR‐TEM images showed the bulk morphology with crystalline lattice, which was consistent with the PXRD experimental results (Figure S10i–l, Supporting Information).

### 2.4 Crystallization Kinetics Analysis

The non‐isothermal crystallization kinetics of **EtP5** *α* was explored by analyzing DSC curves at different heating rates. It was found that the exothermic peak was gradually shifted toward the high temperature region, and the peak width became larger as the heating rate increased (Figure S11a, Supporting Information). The results suggested that **EtP5** *α* could crystallize in the low temperature region at a low heating rate, while **EtP5** *α* tended to crystallize in the high temperature region at a high heating rate. The obtained *X*(t) curve exhibited sigmoidal (*S*‐shaped) behavior (Figure S11b, Supporting Information). It was shown that the crystallinity curve became narrower with increasing the cooling rate, suggesting that **EtP5** *α* could complete crystallization in a short time. In the late stage of crystallization, the increase in crystallinity slowed down significantly, since the mutual contact of grown grain suppressed their further growth. Additionally, **EtP5** *α* exhibited temperature dependence with *S*‐shapes at different heating rates, and the curves shifted to higher temperatures with increasing heating rate due to the occurrence of thermally driven processes (Figure S11c, Supporting Information). The results showed that the nucleation rate of **EtP5** *α* was slower in the initial stage of crystallization, faster in the middle stage of crystallization, and slower in the late stage of crystallization.

The kinetic parameters of non‐isothermal crystallization could be calculated according to the Avrami equation. The *n* (Avrami index) of **EtP5** *α* was above 2.5, indicating that the crystallization was mainly determined by a diffusion‐controlled 3D growth with an increasing nucleation rate (Figure S11d, Supporting Information). Additionally, *n* was not an integer, which showed that homogeneous nucleation and heterogeneous nucleation occurred simultaneously during the crystallization process. The rate constant increased with the increase of the heating rate, indicating that the rapid cooling could speed up the crystallization rate and shorten the crystallization process.

### 2.5 Infrared Thermal Imaging Analysis

**Figure 3:**
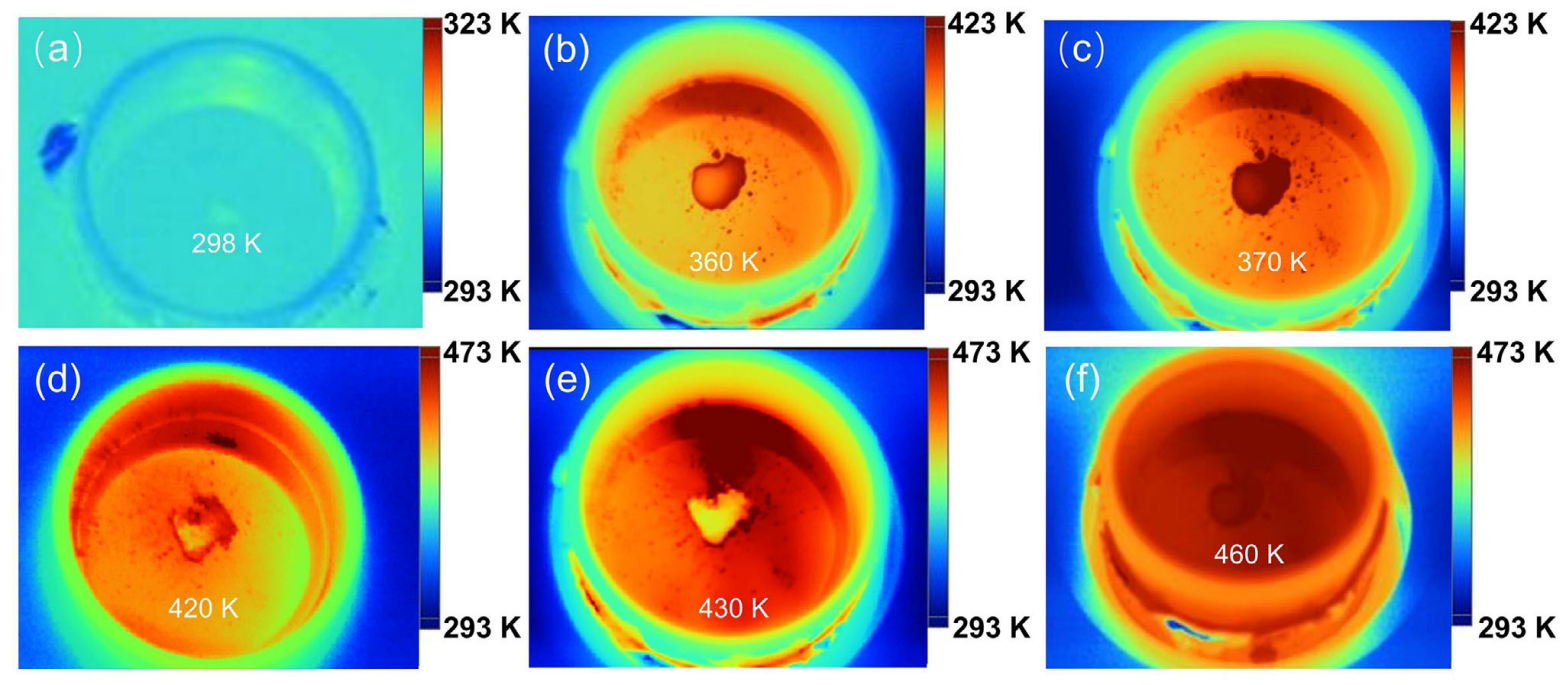
Infrared thermography images of **EtP5** *α* upon heating.

The thermal response caused by the phase transition of **EtP5** *α* upon heating can also be reflected by the temperature change recorded using an infrared thermography camera. In the infrared thermographic image, the red part represented the high temperature and the blue part meant the low temperature. In the initial stage of heating, **EtP5** *α* showed a similar temperature to the ambience, and the image was dominated by blue (**Figure** **3**a). As heated to ≈360 K, the temperature of **EtP5** *α* was higher than the ambient temperature because the phase change from the amorphous state to **EtP5** *α* released heat (Figure 3b,c). As the sample was further heated to ≈425 K, **EtP5** *α* started to melt and absorbed heat from the surrounding environment, thus causing an apparent temperature drop of **EtP5** *α* compared with the ambient temperature (Figure 3d–f). These results directly demonstrated the ability of **EtP5** *α* to store and controllably release thermal energy.

### 2.6 Cycling Stability and Thermal Energy Long‐Term Storage

**Figure 4:**
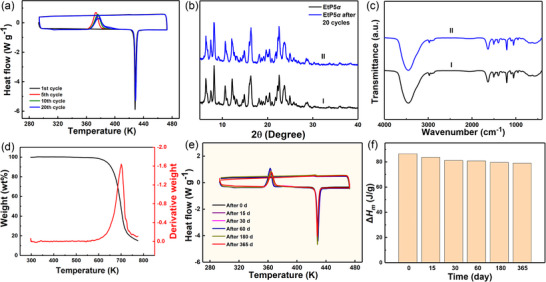
a) DSC curves of **EtP5** *α* at different cycles. b) PXRD patterns of: I) original **EtP5** *α*; II) **EtP5** *α* after 20 cycles. c) FTIR spectra of **EtP5** *α* before and after 20 cycles. d) TGA and corresponding DTG curves of **EtP5** *α*. DSC curves e) and Δ*H*_m_ f) of **EtP5** *α* before and after storage for a period of time.

Excellent cycling stability is highly desired for PCMs to fulfill the requirements of high frequency use in the practical application.^[^ ^70^ ^]^ The DSC curves showed that there was little change in the thermal energy storage performance of **EtP5** *α* during 20 thermal cycles (**Figure** **4**a), and the Δ*H*_m_ value decreased slightly to ≈1.3 J g^−1^. The PXRD patterns and FTIR spectra of **EtP5** *α* before and after 20 cycles indicated that the chemical structure of **EtP5** *α* was very stable during thermal cycles, which laid the foundation for its good cycling stability (Figures 4b,c). Based on such excellent cycling stability of phase change behavior, **EtP5** *α* had potential in the field of thermal energy storage and utilization.

Thermal stability is also an important parameter to evaluate the applicability of PCMs.^[^ ^71^ ^]^ The TGA and corresponding derivative thermogravimetry (DTG) curves of **EtP5** *α* showed negligible weight loss (less than 2%) below 500 K (Figure 4d). These results meant that the melted **EtP5** *α* did not volatilize even as the environment temperature was increased up to 500 K, which greatly widened its operating temperature range.

In industry, thermal energy long‐term storage is one of the potential and key solutions to solve the mismatch between thermal energy supply and demand, which is also the essential property of PCMs.^[^ ^72^, ^73^ ^]^ **EtP5** *α* could remain supercooled state at room temperature or even lower temperature, and store thermal energy for long periods of time. In the absence of external energy input, latent heat would always be stored without releasing, thus achieving the purpose of latent heat long‐term storage (Figure S12, Supporting Information). Moreover, the cold crystallization of **EtP5** *α* was easily triggered by heating, releasing the stored latent heat. After being stored for 365 days, **EtP5** *α* were able to release 91.3% of the latent heat within the temperature range of 350–370 K through inducing the cold crystallization (Figure 4e,f). Compared to other PCMs, **EtP5** *α* exhibited advantages such as controllable heat release, long heat storage time, and simple preparation (Table S1).

### 2.7 Mechanism Analysis of Thermal Energy Storage and Release

**Figure 5:**
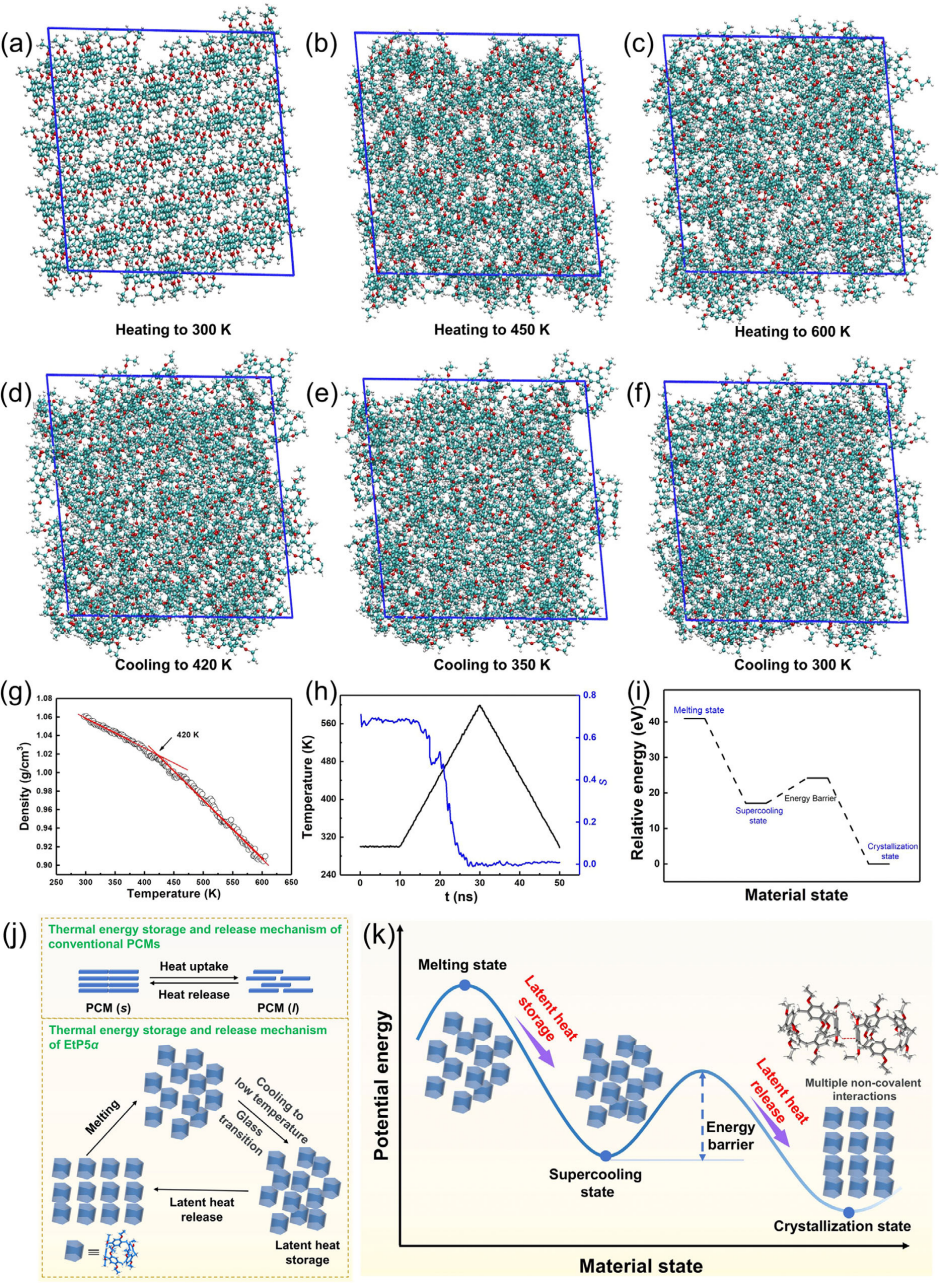
a‐f) Simulation diagram of EtP5α during heating and cooling processes. g) Density of EtP5α at different temperatures. h) Order degree s) of EtP5α during heating and cooling processes. i) Relative energy of EtP5α in different states. j) The mechanism of thermal energy storage and release of conventional PCMs and EtP5α. k) Schematic illustration of thermal energy storage and controllable release of EtP5α.

Molecular dynamics (MD) simulation has emerged as a key tool for material design and performance prediction.^[^ ^74^ ^]^ The use of MD simulation is of great significance to reduce the experimental cost and reveal the mechanism behind phase transitions.^[^ ^75^ ^]^ To gain deep insight into the mechanism of such controllable and reversible thermal energy storage and release behavior of **EtP5** *α*, density functional tight‐binding molecular dynamics (DFTB‐MD) simulations were performed based on the crystal structure of **EtP5** *α*. As the temperature was increased to above 450 K, **EtP5** *α* drastically transformed to the disordered state because the multiple non‐covalent interactions between **EtP5** *α* molecules were destroyed by the accelerated molecular motion (VideoS1, Supporting Information and **Figure** **5**a–c). However, during the cooling process, the temperature drop did not significantly alter the disordered distribution of **EtP5** *α* molecules. Even as the temperature was lowered down to 300 K, **EtP5** *α* were still in highly disordered state, suggesting that the multiple non‐covalent interactions between **EtP5** *α* molecules did not recover (Figure 5d–f). DFTB‐MD simulations indicated that the glass transition temperature (*T_g_*) of **EtP5** *α* was ≈420 K (Figure 5g), which was consistent with the DSC results. Thereby, the heat absorbed during the melting process was well stored in the supercooled **EtP5** *α* at a temperature below 420 K. The change of order degree (*s*) of **EtP5** *α* during heating and cooling processes was simulated (Figure 5h), showing that the *s* values of the original and supercooled **EtP5** *α* were 0.71 and 0.02, respectively. In addition, the intermolecular energy results confirmed that the multiple noncovalent interactions between **EtP5** *α* molecules gradually weakened during the heating process (Figure S13, Supporting Information).

Combined with first‐principles molecular dynamics calculations, the energies of **EtP5** *α* in different states were given. The calculation results confirmed the existence of the energy barrier (Figure 5i; Figure S14, Supporting Information). For conventional PCMs, the liquid phase is directly transformed into the initial solid phase upon cooling, accompanied by spontaneous thermal energy release.^[^ ^76^ ^]^ In contrast, due to multiple non‐covalent interactions, the energy barrier for orderly arrangement of **EtP5** *α* was enlarged, which was difficult to be overcome upon cooling process (Figure 5j). Therefore, the amorphous supercooled phase was stabilized and the absorbed thermal energy was well retained at room temperature. To induce the crystallization of the supercooled **EtP5** *α*, additional energy input was needed to overcome the energy barrier. As a consequence, the controllable thermal energy release could be achieved through heating (Figure 5k). In addition, through manipulating the energy barrier by appropriate structural design or activated treatment, switchable and controllable thermal energy release can be realized in a desired temperature range.

### 2.8 Thermal Energy Switchable Release

**Figure 6:**
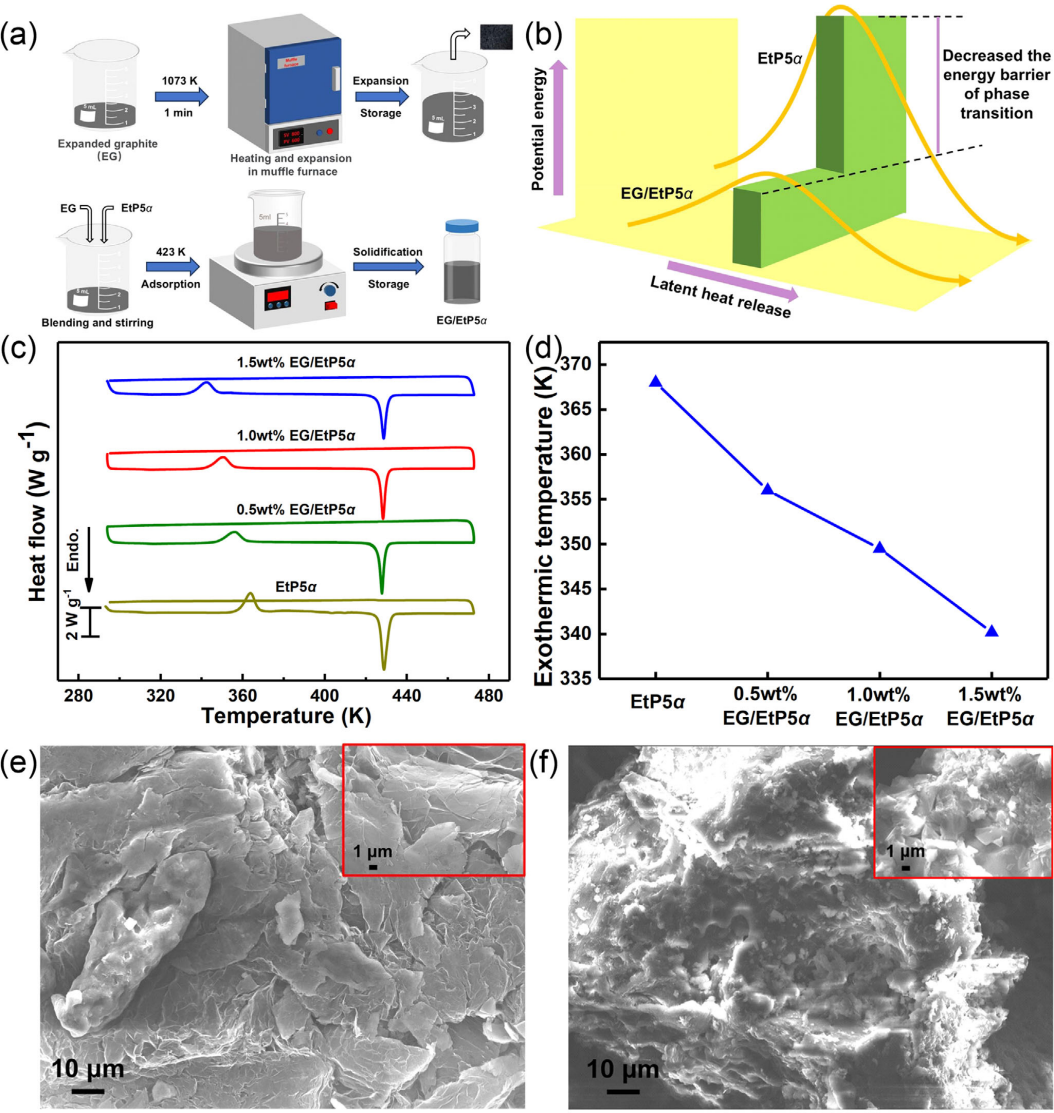
a) The preparation process of expanded graphite (EG) and EG**/EtP5** *α*. b) Schematic illustration of loading EG to reduce the energy barrier of EG**/EtP5** *α*. DSC curves c) and exothermic temperatures d) of **EtP5** *α*, 0.5wt% EG/**EtP5** *α*, 1.0wt% EG/**EtP5** *α* and 1.5wt% EG/**EtP5** *α*. SEM images of EG e) and 1.5wt% EG/**EtP5** *α* f).

From the prospective of crystallization, lowering the energy barrier can also be realized through promoting the heterogeneous nucleation.^[^ ^77^ ^]^ Doping expanded graphite (EG) into **EtP5** *α* is a promising method because the large specific surface area of EG provides a large number of nucleation sites for **EtP5** *α*. The N_2_ isothermal adsorption and desorption experiments demonstrated that EG had a large specific surface area and pore volume of 138.885 m^2^ g^−1^ and 0.222 cm^3^ g^−1^, respectively (Figure S15, Supporting Information). Due to capillary force and surface tension, **EtP5** *α* were easily adsorbed into the EG pore and adhered to the graphite sheet (**Figure** **6**a). By simply changing the loading amount of EG to control the energy barrier, the phase transition temperature of the composite PCM (EG**/EtP5** *α*) can be further adjusted to meet the requirements of use under different conditions (Figure 6b).

The phase transition characteristics of EG**/EtP5** *α* with different EG mass fractions were measured by DSC. It was found that the exothermic temperatures of EG/**EtP5** *α* were reduced compared with **EtP5** *α* (Figure 6c). Through loading EG with different mass fractions, the exothermic temperatures of EG/**EtP5** *α* could be adjusted from 368 to 340.2 K, which indicated that the exothermic temperature gradually decreased as the EG loading amount increased (Figure 6d). The 1.5wt% EG/**EtP5** *α* showed the lowest exothermic temperature, but still exhibited excellent thermal energy controllable release. During 20 thermal cycles, the high thermal energy storage capacity of 1.5wt% EG/**EtP5** *α* could also be maintained (Figure S16, Supporting Information). After doping with EG, the thermal conductivity of 1.5wt% EG/**EtP5** *α* was improved to 0.19 W mK^−1^. Moreover, the morphology of EG was vermicular, consisting of overlapping and intersecting graphite flakes with a large number of gaps where PCMs could be adsorbed (Figure 6e; Figure S17, Supporting Information). **EtP5** *α* were loaded on the lamellar EG with a relatively dense structure, suggesting that **EtP5** *α* were successfully adsorbed into the pores of EG (Figure 6f). Due to the good compatibility of **EtP5** *α* with EG, **EtP5** *α* still maintained the original blocky structure. Moreover, in the FTIR spectra of 1.5wt% EG/**EtP5** *α*, it was observed that no new functional groups of EG and **EtP5** *α* appeared, indicating that there was no chemical reaction between EG and **EtP5** *α*, only physical doping (Figure S18, Supporting Information).

## 3 Conclusion

In summary, we discover for the first time that activated **EtP5** (**EtP5** *α*) are excellent PCMs that can be used for thermal energy long‐term storage as well as controllable and switchable release. Multiple non‐covalent interactions between **EtP5** *α* molecules allow them to storage latent heat stably at room temperature or lower temperature for more than 365 days. Then the supercooled **EtP5** *α* can be thermally induced to crystallize, achieving the thermal energy controllable release. In addition, through manipulating the energy barrier, switchable thermal energy release can be realized in a desired temperature region. As PCMs, **EtP5** *α* show high latent heat, controllable and switchable thermal energy release, long heat storage time, excellent cycling stability and thermal stability, which have broad application prospects in the field of thermal energy storage and utilization. Moreover, MD simulations combined with experimental characterizations have confirmed that the thermal energy storage and release process is accompanied by the transformation of ordered **EtP5** *α* to disordered amorphous phase and then to ordered **EtP5** *α*, which provides a new insight into the thermal energy storage and controllable release. This research will give new ideas for the design of new PCMs and the development of advanced energy and heat utilization technology based on PCMs.

## Conflict of Interest

The authors declare no conflict of interest.

## Supporting information



Supporting Information

Supplemental Video 1

## Data Availability

The data that support the findings of this study are available from the corresponding author upon reasonable request.
