# Supplementary material for: Supramolecular Phase Change Materials for Spatiotemporally Thermal Energy Utilization
Source: Adv Sci (Weinh). 2025 Dec 12;13(6):e12924. doi: 10.1002/advs.202512924 (PMC12866706; doi:10.1002/advs.202512924)
Supplement: Supplementary file 1 — Supporting Information [file ADVS-13-e12924-s001.docx]

**Supramolecular Phase Change Materials for Spatiotemporally Thermal Energy Utilization**

Miaomiao Yan,^1+^ Cong Liu,^2+^ Ruolan Tang,^2^ Xueyu Zhu,^1^ Zongbin Li,^2*^ and Jiong Zhou^1*^

*^1^Department of Chemistry, College of Sciences, Northeastern University, Shenyang 110819, China.
^2^Key Laboratory for Anisotropy and Texture of Materials (Ministry of Education), School of Materials Science and Engineering, Northeastern University, Shenyang 110819, China.*

*Email: lizb@atm.neu.edu.cn;* [*zhoujiong@mail.neu.edu.cn*](mailto:zhoujiong@mail.neu.edu.cn)

*[*+*] Both authors contributed equally to this work.*

**Supporting Information (17 Pages)**

| 1. | *Materials* | S2 |
| --- | --- | --- |
| 2. | *Methods* | S2 |
| 3. | *Characterization of* ***EtP5****α* | S4 |
| 4. | *Thermal Energy Storage* | S5 |
| 5. | *Crystallization Kinetics Analysis* | S8 |
| 6. | *Computational Details* | S11 |
| 7. | *Thermal Energy Switchable Release* | S14 |
| 8. | *References* | S16 |

1. *Materials*

Perethylated pillar[5]arene (**EtP5**) were synthesized as described previously.^1^ Activated crystalline **EtP5** were referred to as **EtP5***α*. **EtP5***α* were prepared according to the reported procedure.^2^ **EtP5***α* were stored in a sealed container in the laboratory (with a temperature range of 288 K to 303 K), away from vibration and impurities.

*2. Methods*

*2.1. Solution NMR*

Solution ^1^H NMR spectra were recorded at 600 MHz using a Bruker Avance 600 NMR spectrometer.

*2.2. Powder X-ray Diffraction*

Powder X-ray diffraction (PXRD) data were collected on a Rigaku Ultimate-IV X-ray diffractometer operating at 40 kV/30 mA using the Cu K*α* line (*λ* = 1.5418 Å). Data were measured over the range of 5−45° in 5°/min steps over 8 min.

*2.3. Thermogravimetric Analysis*

Thermogravimetric analysis (TGA) was carried out using a Q5000IR analyzer (TA Instruments) with an automated vertical overhead thermobalance. The samples were heated at 10 K/min using N_2_ as the protective gas.

*2.4. Fourier Transform Infrared Spectra*

Fourier Transform Infrared (FT-IR) spectra were recorded on a VERTEX70 FT-IR spectrophotometer operating at 32 scans and 2 cm^-1^ resolution in the region between 4000 and 400 cm^-1^ at room temperature. The dried **EtP5***α* samples were mixed with KBr at a mass ratio of 1:50, and then pressed into thin sheets. The thin sample was placed in the light path of the FTIR spectrometer. In situ FT-IR spectra were carried out using the German Bruker V70.

*2.5. Differential Scanning Calorimetry Analysis*

The phase change behaviors of **EtP5***α* and EG**/EtP5***α* were measured by differential scanning calorimetry (DSC, TA-Discovery DSC25). A 5-10 mg sample was placed in the DSC sample cell and tested at a heating/cooling rate of 10 K/min in a temperature range of 293−473 K. The phase transition temperatures and phase transition enthalpy were obtained. Additionally, we transferred **EtP5***α* to the ultrasonic device for triggering tests. The thermocouple was inserted into the molten sample, and then the temperature of the sample was gradually cooled down to room temperature, forming the supercooled sample. After stabilizing at room temperature for a certain period, the crystallization and exothermic processes were triggered by activating the ultrasonication (700 W).

*2.6. Polarizing Optical Microscope*

The melting and crystallization behaviors of **EtP5***α* during thermal cycles were tested by a POM instrument (POM, Leica EP 50). The sample was made into transparent small pieces, placed on the hot stage, heated and cooled at the rate of 2 K/min, and tested in the range of 293 ~ 473 K to observe the changes of **EtP5***α*.

*2.7. Infrared Thermal Imager Analysis*

**EtP5***α* were placed on a hot stage. The temperature distribution and temperature change of **EtP5***α* were recorded by the infrared thermal camera (Fluke Ti27).

*2.8. Morphological Features*

The morphological features of **EtP5***α* were characterized by JEOL JSM-7001F scanning electron microscope (SEM) and EOL JEM-2100F transmission electron microscope (TEM).

*3. Characterization of* ***EtP5****α*


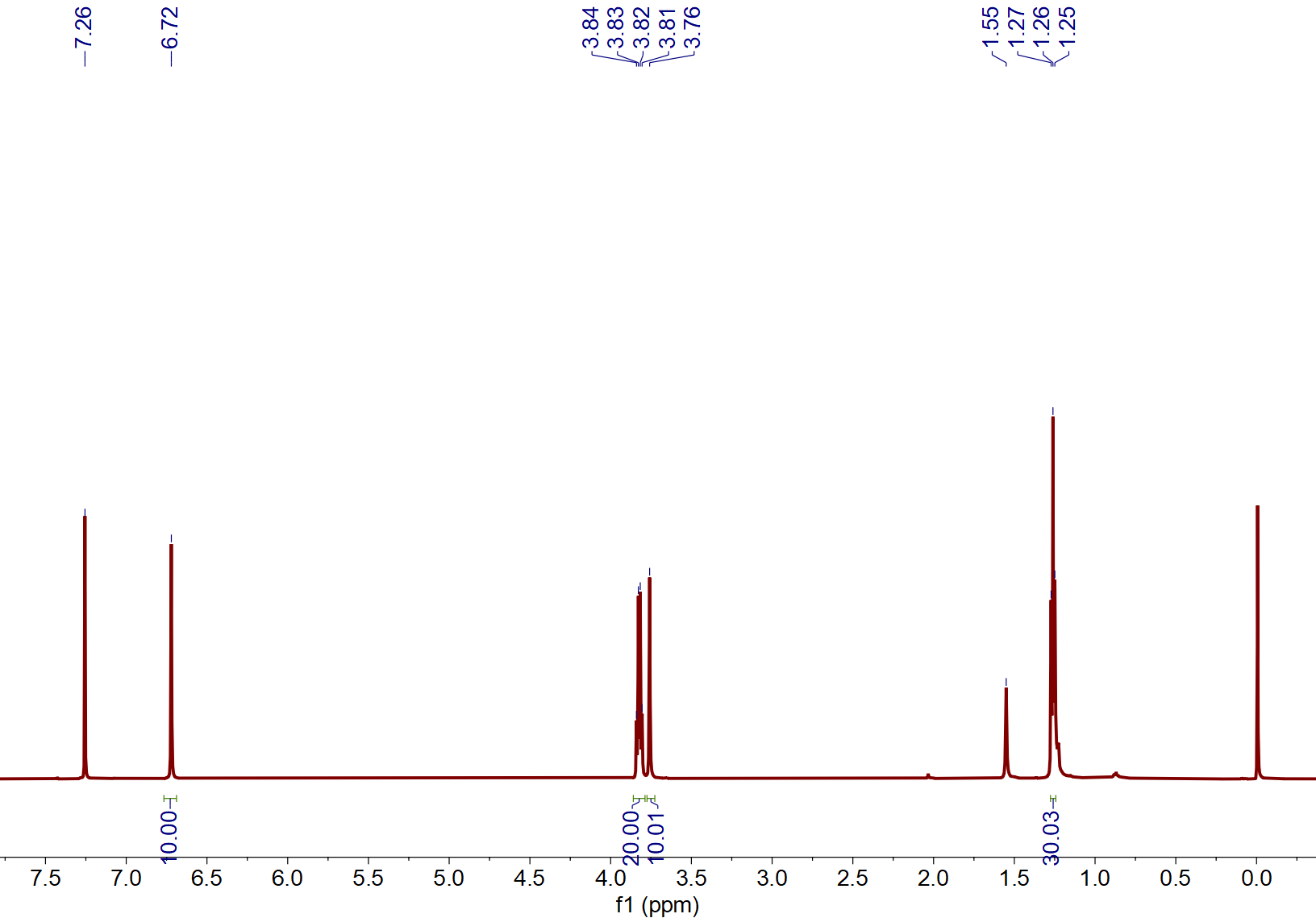


**Figure S1.** ^1^H NMR spectrum (600 MHz, CDCl_3_, 293 K) of **EtP5***α*.


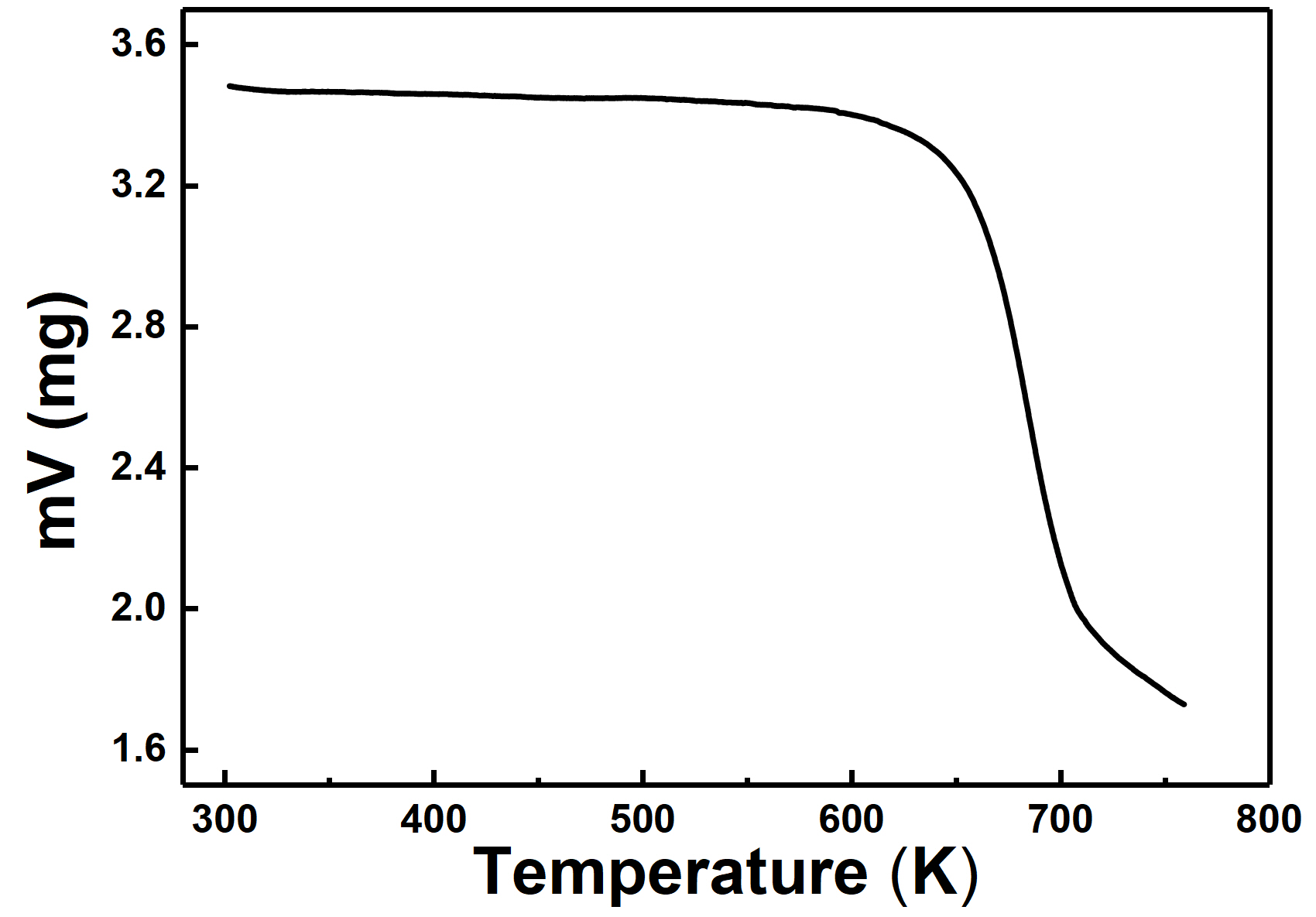


**Figure S2.** Thermogravimetric analysis of **EtP5***α*.

*
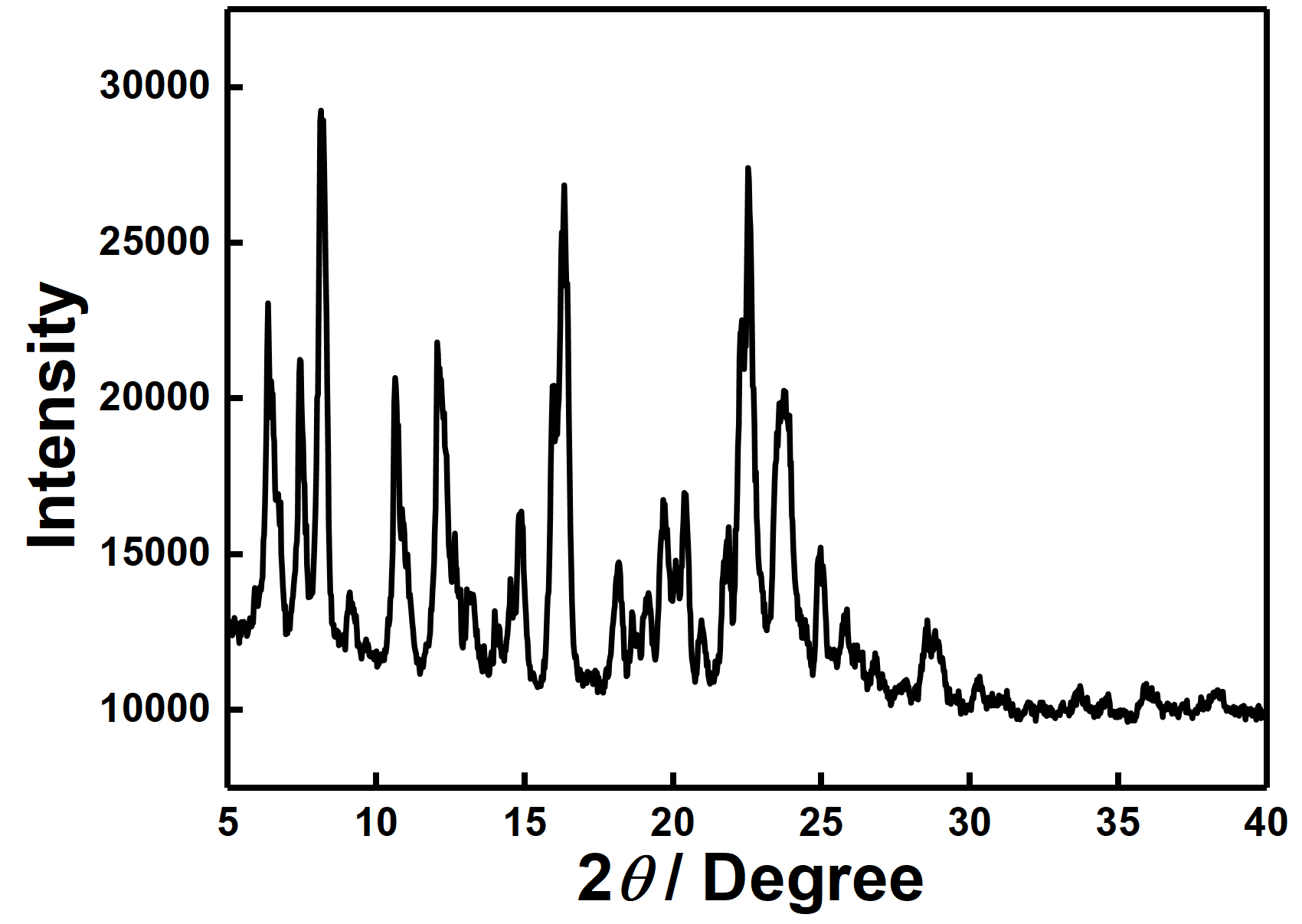
*

**Figure S3.** Powder X-ray diffraction pattern of **EtP5***α*.

*4. Thermal Energy Storage*


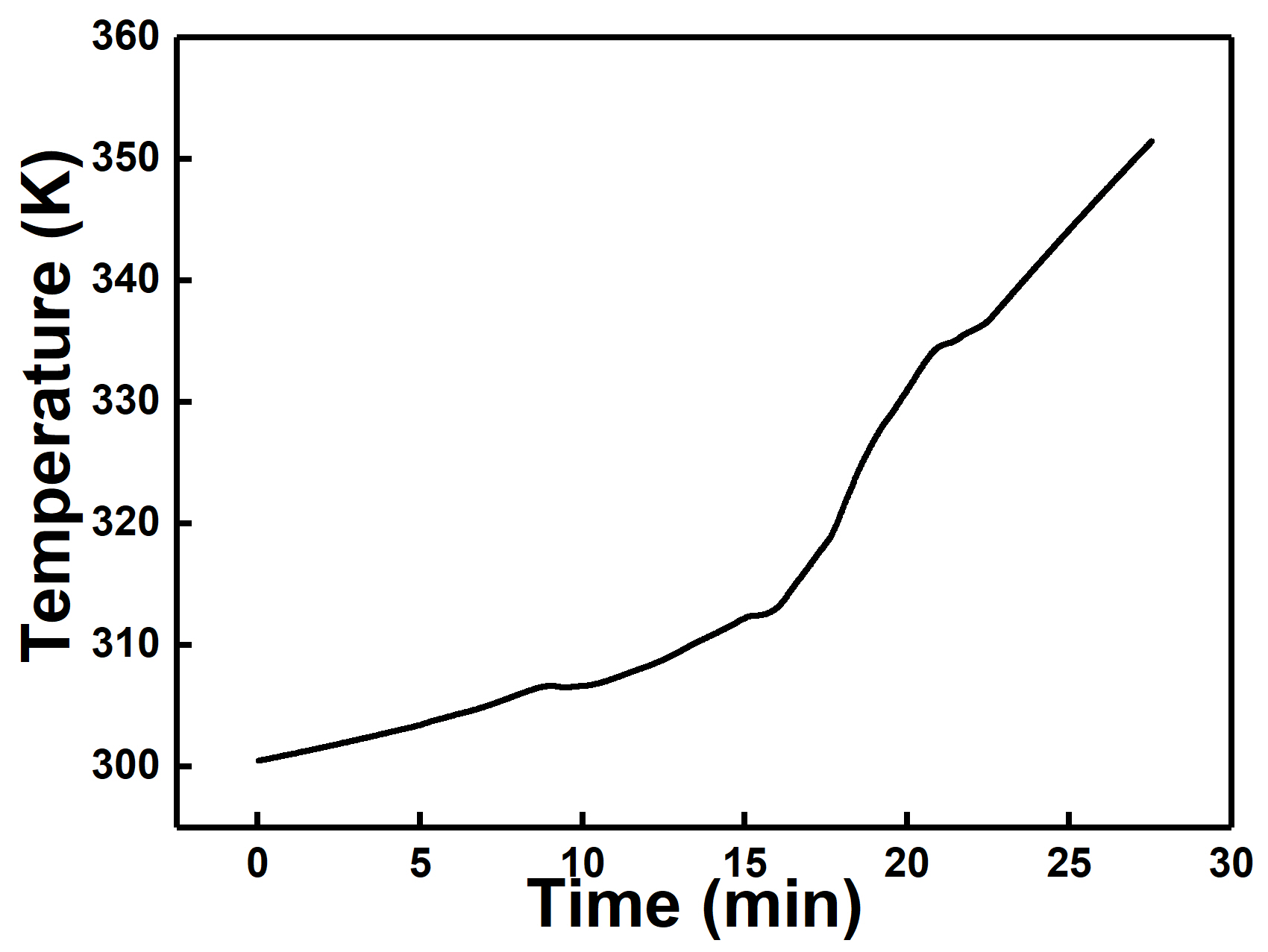


**Figure S4.** Temperature changes of supercooled **EtP5***α* after ultrasonication.


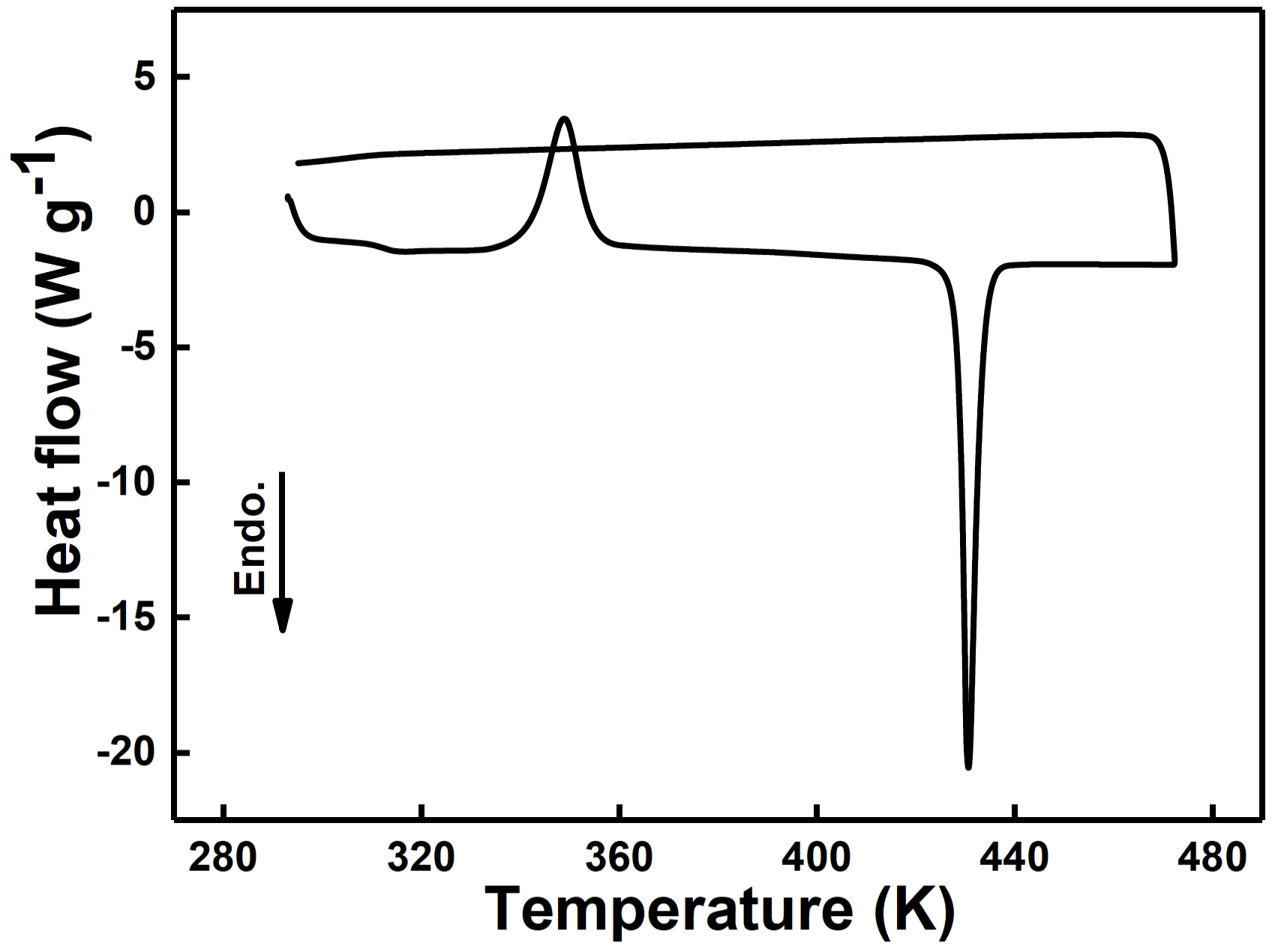


**Figure S5.** DSC curves of **EtP5***α* after ultrasonication.


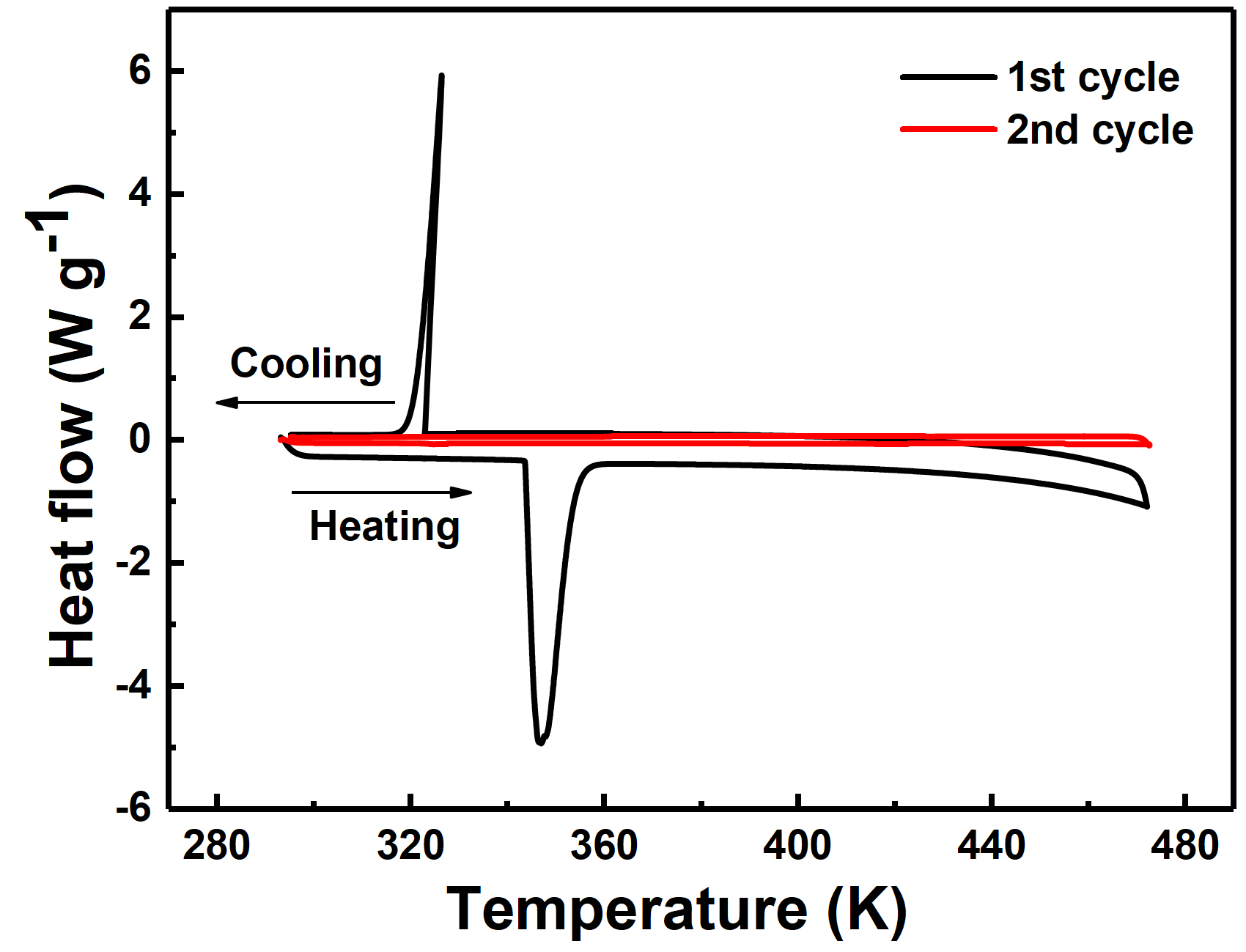


**Figure S6.** DSC curve of *p*-diethoxybenzene.


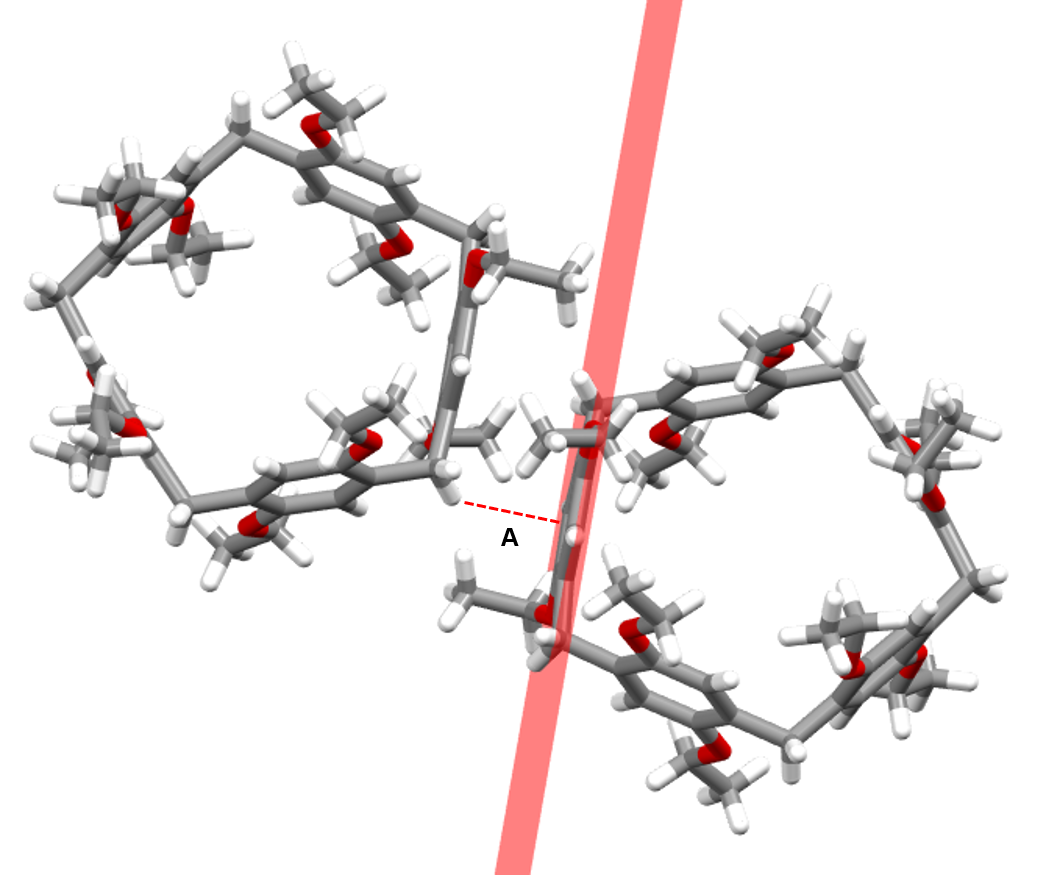


**Figure S7.** Schematic illustration of C−H···π interaction between **EtP5***α* molecules. H−*π*-plane distance: A = 2.885 Å.


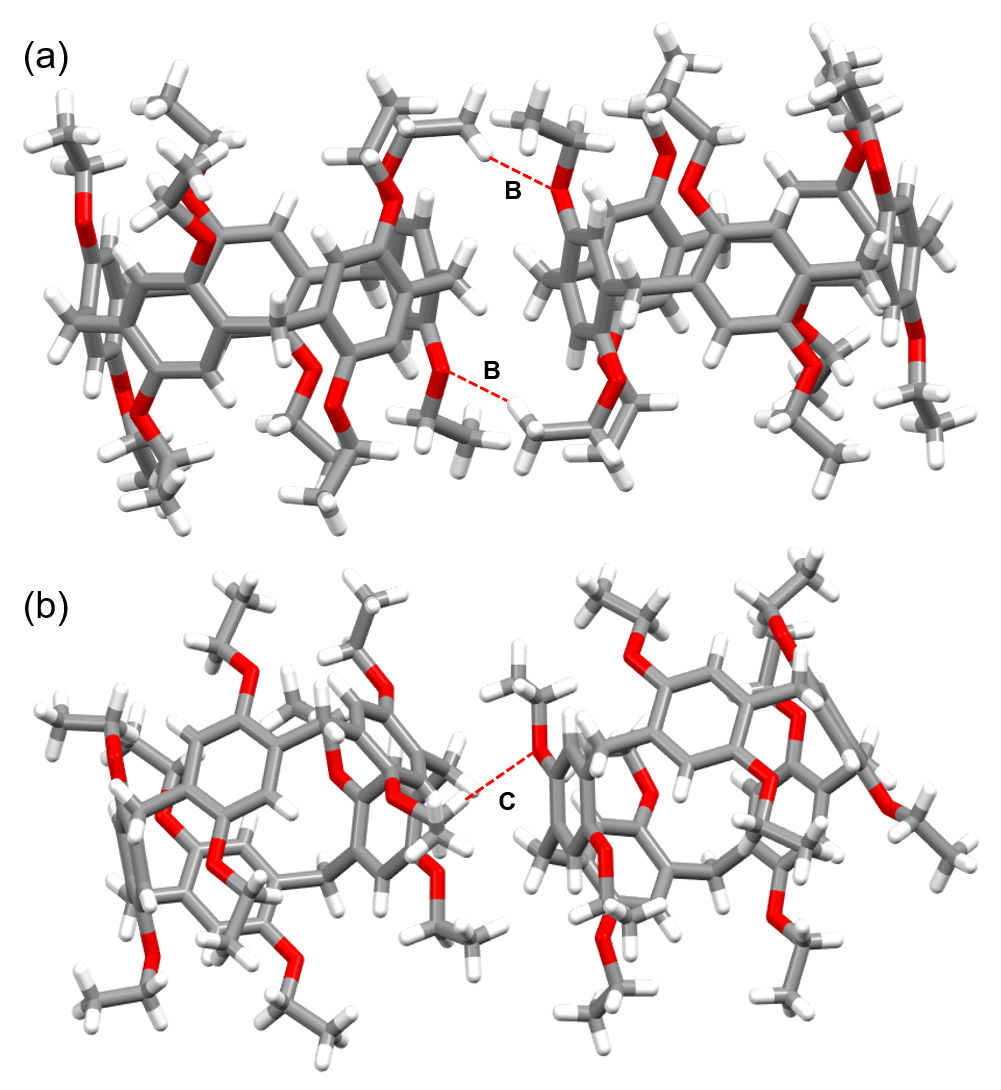


**Figure S8.** Schematic illustration of C−H···O interaction between **EtP5***α* molecules. H−O distances: B = 2.656 Å, C = 2.840 Å.


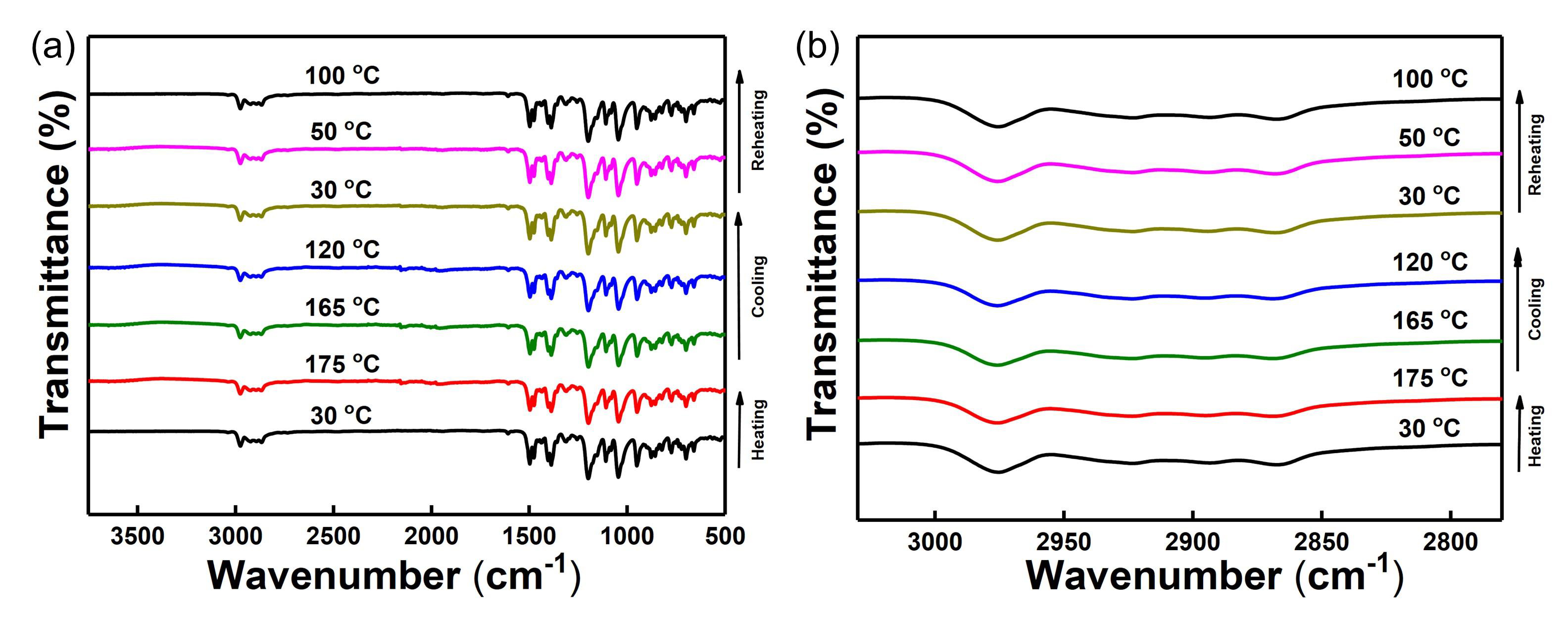


**Figure S9.** In situ infrared spectra: (a) **EtP5***α* at different temperatures; (b) the partial view of (a).


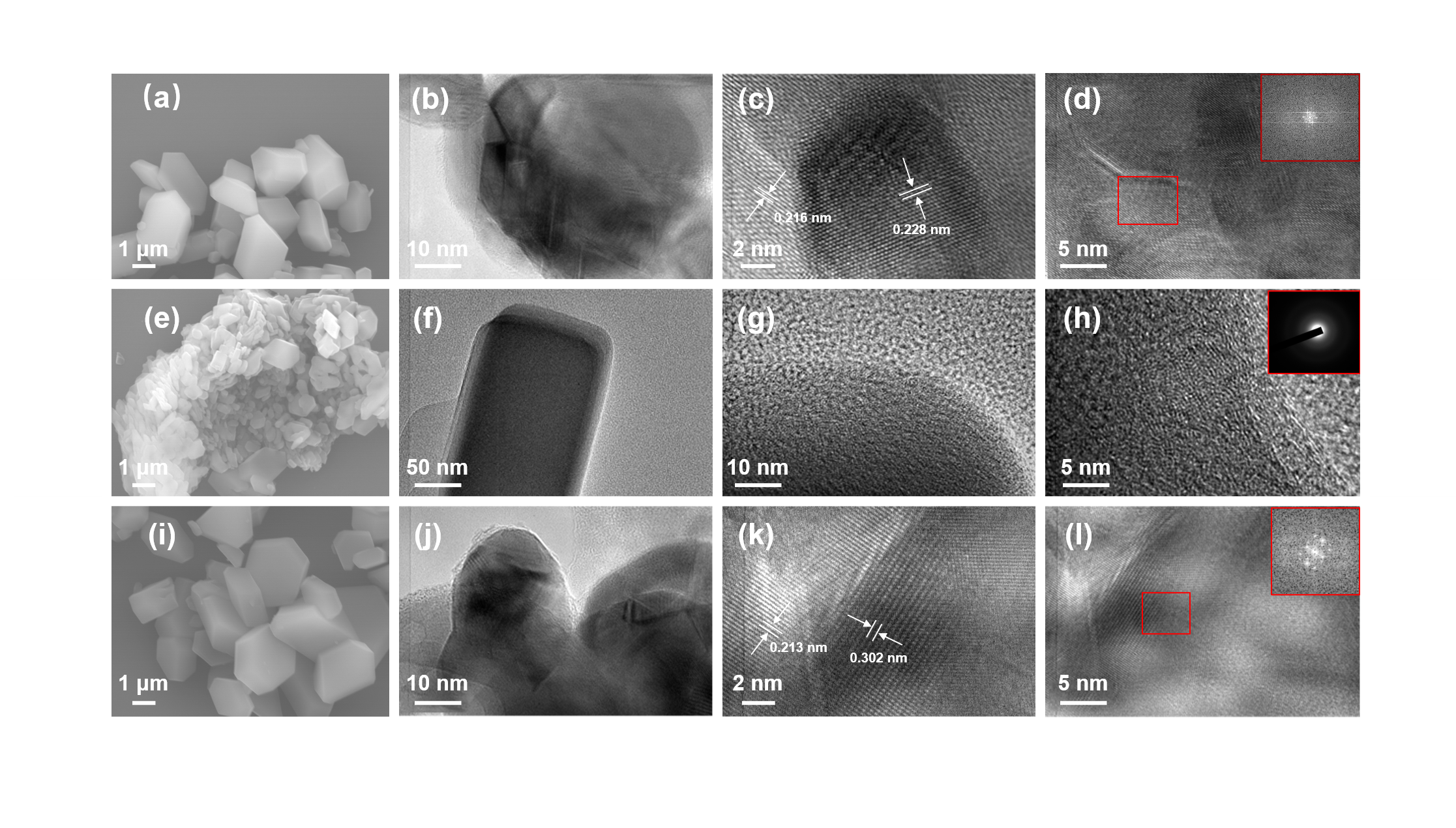


**Figure S10.** SEM and HR-TEM images of **EtP5***α* in thermal energy storage and release. (a-d) Original **EtP5***α*. (e-h) **EtP5***α* after glass transition. (i-l) Reheated **EtP5***α* after glass transition. (The inserts showed corresponding selected area electron diffraction pattern).

*5.* *Crystallization Kinetics Analysis*

The non-isothermal crystallization kinetics of **EtP5***α* was explored by analyzing DSC curves at different heating rates. The DSC curves of **EtP5***α* at different heating rates were integrated according to equations (1) and (2). The relevant equations utilized in the kinetics determination are as follows:

*X*(t) = $\frac{\int_{\text{T}\text{0}}^{\text{T}} \text{(dHc/dt)dt}}{\int_{\text{T}\text{0}}^{\text{T}\text{e}} \text{(dHc/dt)dt}}$ (1)

*X*(T) = $\frac{\int_{\text{T}\text{0}}^{\text{T}} \text{(dHc/dT)dT}}{\int_{\text{T}\text{0}}^{\text{T}\text{e}} \text{(dHc/dT)dT}}$ (2)

Where *T*_0_ and *T*_e_ designate the starting and ending temperature of crystallization, respectively. The *dH_c_*/*dt* denotes the enthalpy of crystallization generated per unit temperature and time. The t, T, and *X* represent time, temperature, and relative crystallinity, respectively.

The kinetic parameters of non-isothermal crystallization could be calculated according to the Avrami equation. The lgt ~ lg[-ln(1−X(t))] relationship curve was calculated from Eq (3) and Eq (4). The relevant equations used in the kinetic determination are as follows:

lg[-ln(1−X(t))] = lg*Z* + *n* lgt (3)

lg*Z_c_* = (lg*Z*)/*β*  (4)

Where *β* denotes the temperature rise rate. *Z* is the crystallization rate constant, and *n* is the Avrami index.


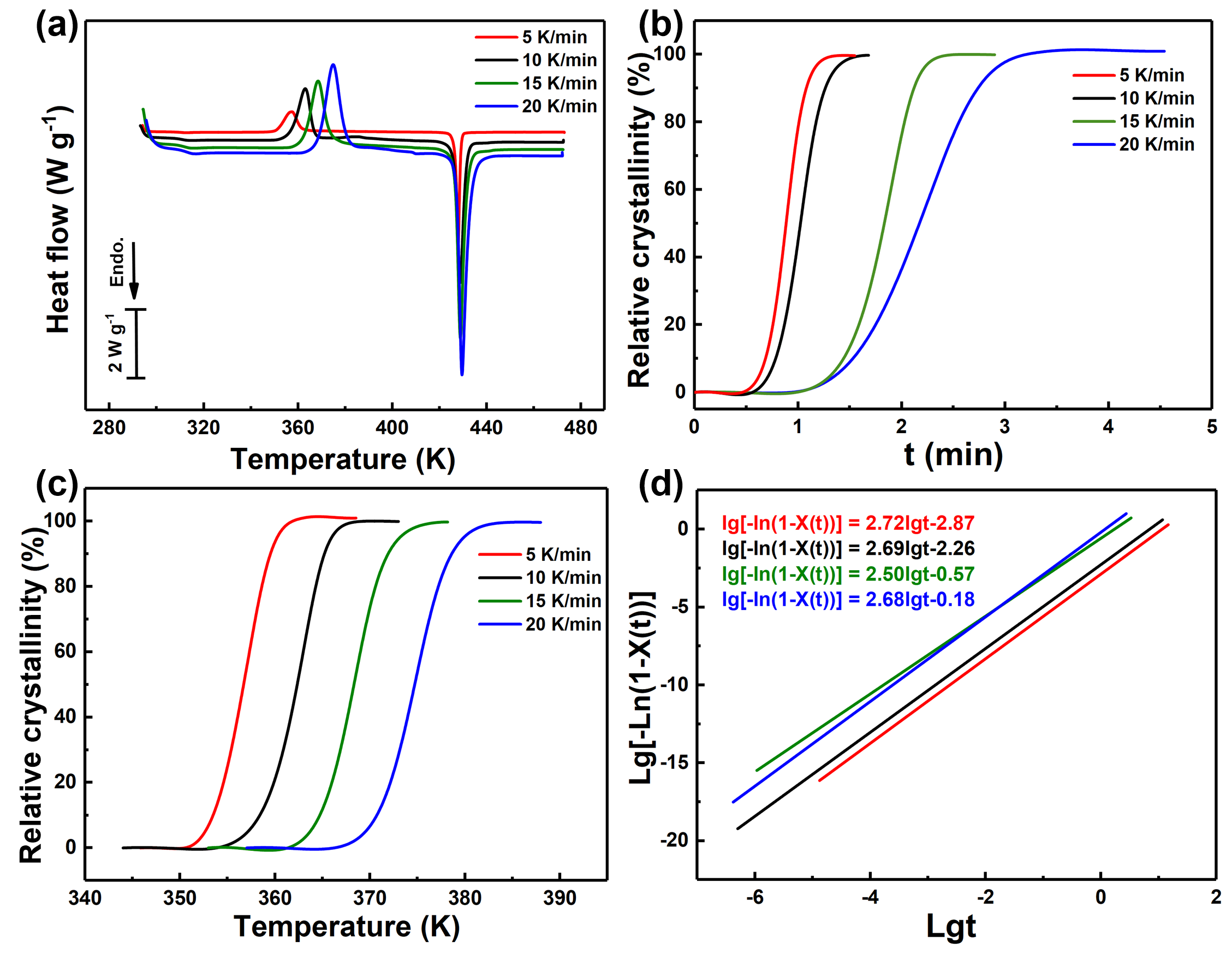


**Figure S11.** (a) DSC curves of **EtP5***α* at different heating rates. (b and c) The relative crystallinity of **EtP5***α* at different heating rates varies with time and temperature. (d) Avrami plots of **EtP5***α* at different heating rates.


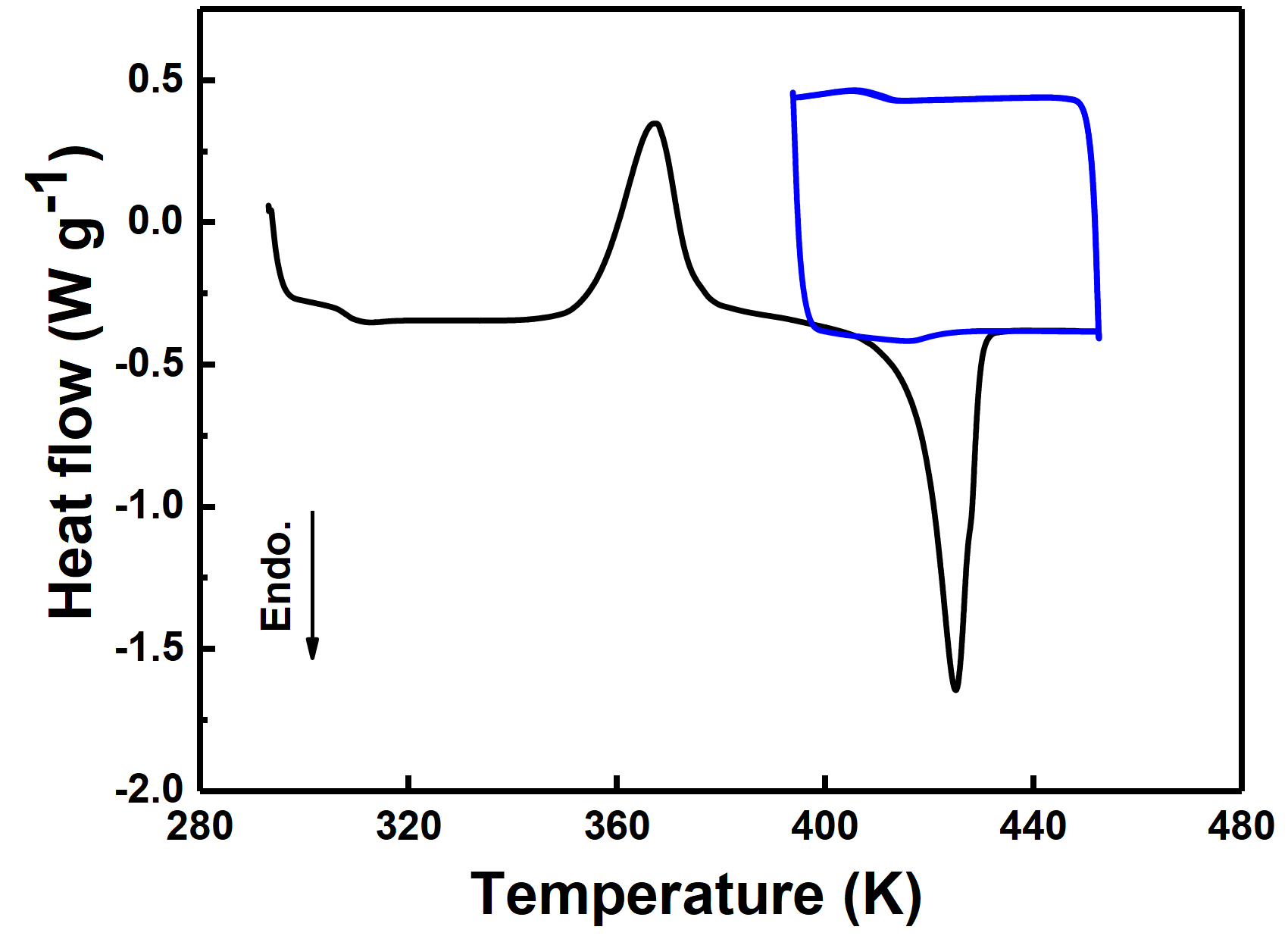


**Figure S12.** DSC curve of **EtP5***α*. The black line indicated that latent heat release could only be triggered during the heating stage, and the blue line showed that the latent heat was stored during the high temperature stage.

**Table S1.** Comparison of phase transition properties of **EtP5***α* with reported typical PCMs.

| Traditional PCMs | Material | ΔH_m_  (J/g) | Subcooling degree | Cycle number, ΔHₘ retention rate (%) | Heat storage time | Advantages and disadvantages | Ref. |
| --- | --- | --- | --- | --- | --- | --- | --- |
|  | Erythritol | 198 | 60 ℃ | 5 cycle (56%) | **/** | High enthalpy change, unstable supercooling, uncontrollable heat release | [3, 4] |
|  | Sodium acetate trihydrate | 258 | More than 138 ℃ | 50 cycle (86%) | **/** | High enthalpy change, large supercooling, easy phase separation, uncontrollable heat release | [5, 6] |

**Table S1.** (continued)

|  | Material | ΔH_m_  (J/g) | Supercooling degree | Cycle number, ΔHₘ retained after cycle | Heat storage time | Advantages and disadvantages | Ref. |
| --- | --- | --- | --- | --- | --- | --- | --- |
| Traditional PCMs | Polymeric PCMs (S-AA-SE) | 31 | 25 ℃ | 5000 cycle (91%) | / | Low latent heat, good circulation, uncontrollable heat release | [7] |
|  | Metallic PCMs (Ni_49.5_Mn_44.5_  Ti_6_)_99.8_B_0.2_ (at.%) | 65.2 | About 25 ℃ | 1000 cycle (/) | / | Low latent heat and good circulation, uncontrollable heat release | [8] |
| Spatiotemporal PCMs | Erythritol/sodium carboxymethylcellulose/tetrasodium ethylenediaminetetraacetate | 178.1 | High supercooling (no heat release at room temperature but heat release after reheating) | 20 cycle (/) | two months | High enthalpy change, complex preparation, complex preparation, short storage time, controllable heat release | [9] |
|  | Erythritol-based composite materials (ERY-SA-CC) | 181.2 | High supercooling (no heat release at room temperature but heat release after reheating) | 80 cycle (/) | 50 day | High enthalpy change, complex preparation, complex preparation, short storage time, controllable heat release | [10] |
|  | **EtP5***α* | 86.5 | High supercooling (no heat release at room temperature but heat release after reheating) | 20 cycle  (94.5%) | 365 day (91.3%) | Low enthalpy change, Simple and efficient synthesis, long storage time, controllable heat release | Our Work |

*6. Computational Details*

Atomistic molecular dynamics simulations were performed in the GROMACS (version 2020.6) simulation package using the General Amber Force Field (GAFF2).^11^

The interaction potential $V$ between atoms includes the bonded and non-bonded terms.

*V = V_bonded_* + *V_nonbonded_*

The bonded terms include the harmonic oscillation of bonds and angles, as well as the torsional rotation of dihedrals; the nonbonded terms include the electrostatic interactions between atoms with partial charges, as well as the Van der Waals interactions described through the Lennard-Jones 12-6 potentials.

$V_{nonbonded}=\sum_{i=1}^{N} \sum_{j=i+1}^{N} \left\{\frac{q_{i}q_{i}}{4\pi\vartheta_{0}\vartheta_{r}r_{ij}}+4\varepsilon_{ij}\left[\left(\frac{\sigma_{ij}}{r_{ij}}\right)^{12}-\left(\frac{\sigma_{ij}}{r_{ij}}\right)^{6}\right]\right\}$

Where *V_bonded_* is bonded potential energy. The *k_b_* designates the bond-stretching force constant. The *l* and *l_0_* respectively represent the bond length and the average bond length. The *k_θ_* designates the bond angle bending force constant. The *θ* and *θ_0_* respectively represent the bond angle and the average bond angle. The *k_ϕ_* designates the energy barrier height. The *n*, *ϕ*, and *ϕ*_0_ respectively represent the number of torsion cycle, dihedral angle, and equilibrium torsion angle. Additionally, *V_nonbonded_* is nonbonded potential energy. The *q*, *ϑ_0_*, *ϑ_r_*, and *r_ij_* respectively represent atomic charge, vacuum dielectric constant, relative dielectric constant, and interatomic distance. The *ε_ij_* and *σ_ij_* respectively represent the potential well depth and the sum of van der Waals radii between atoms.

The crystal structures of **EtP5***α* were optimized and structural models were established. After 2000 steps of energy minimization, the system was equilibrated under 300 K for 10 ns, followed by a 20 ns heating to the target temperature of 600 K, and then gradually annealed to 300 K within 20 ns. The density and order parameters of the structural model were monitored, and the trajectory was kept every 10 ps. The temperature was controlled using the Nose-Hoover method, and the pressure was regulated to atm using the Parrinello-Rahman method. The cutoff scheme of 1.2 nm was implemented for the non-covalent interactions, and the Particle Mesh Ewald method with a Fourier spacing of 0.1 nm was applied for the long range electrostatic interactions.^12^ All bonds with hydrogen atoms were constrained using the LINCS algorithm.^13^

^
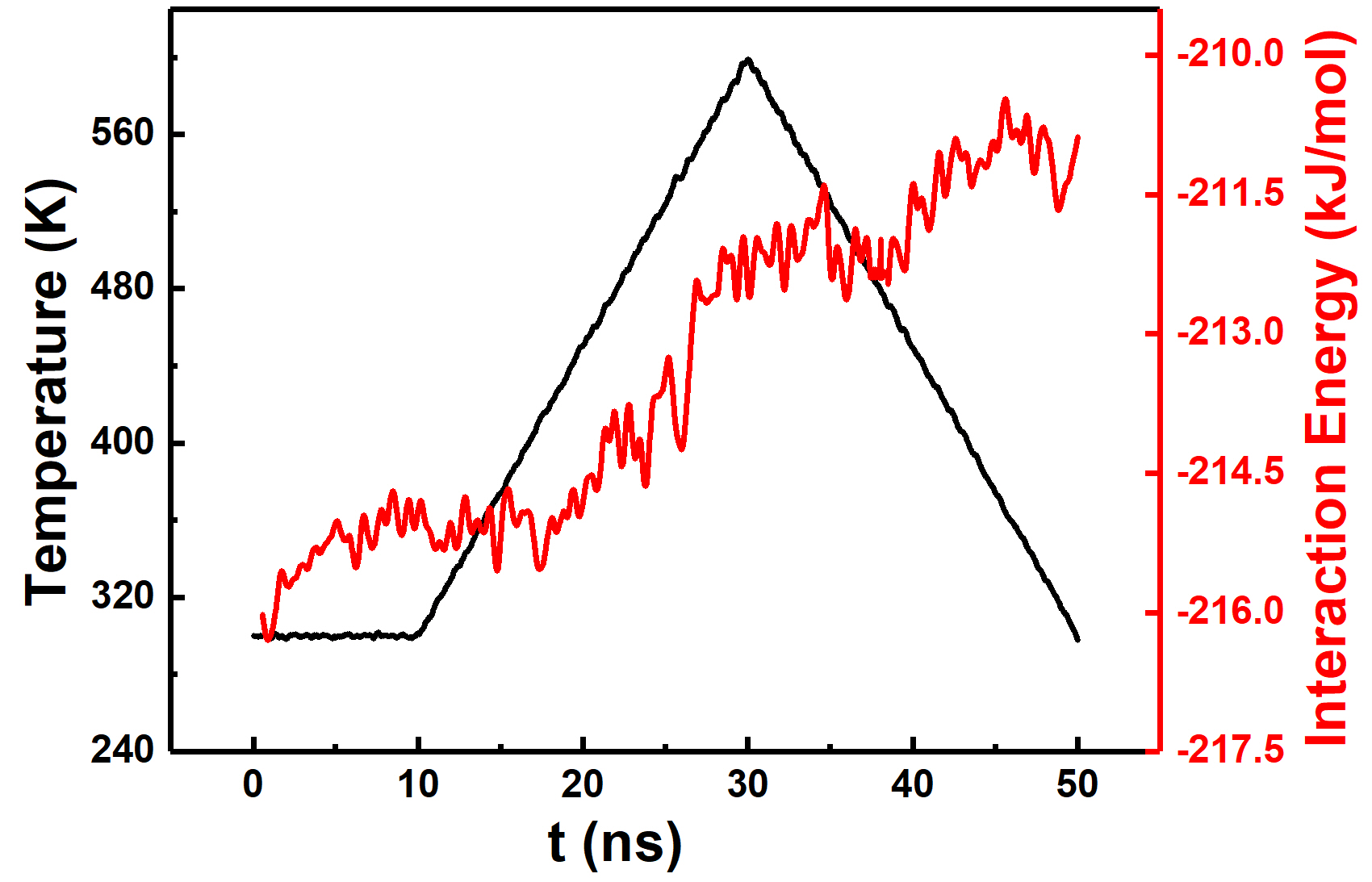
^

**Figure S13.** Simulated non-covalent interaction energy of **EtP5***α* during heating and cooling processes.

First-principles molecular dynamics simulations were performed in the NVT ensemble with a Nose-Hoover bath for temperature control. The initial temperature was set at 273 K, then ramped up to 500 K and then down to 358 K with a time step of 1.0 fs. The calculated structures of **EtP5***α* in different states were shown in Figure S14.


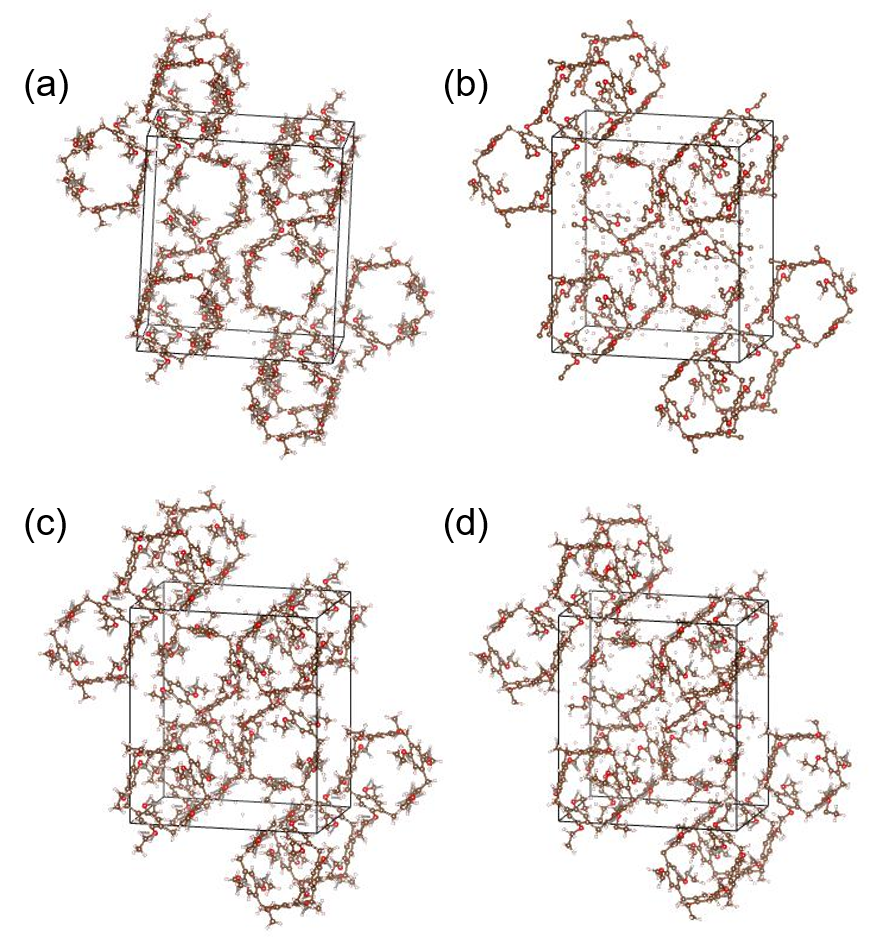


**Figure S14.** Relative energy of **EtP5***α* in different states. (a) Meating state; (b) Supercooling state; (c) Intermediate state; (d) Crystallization state.

*7. Thermal Energy Switchable Release*


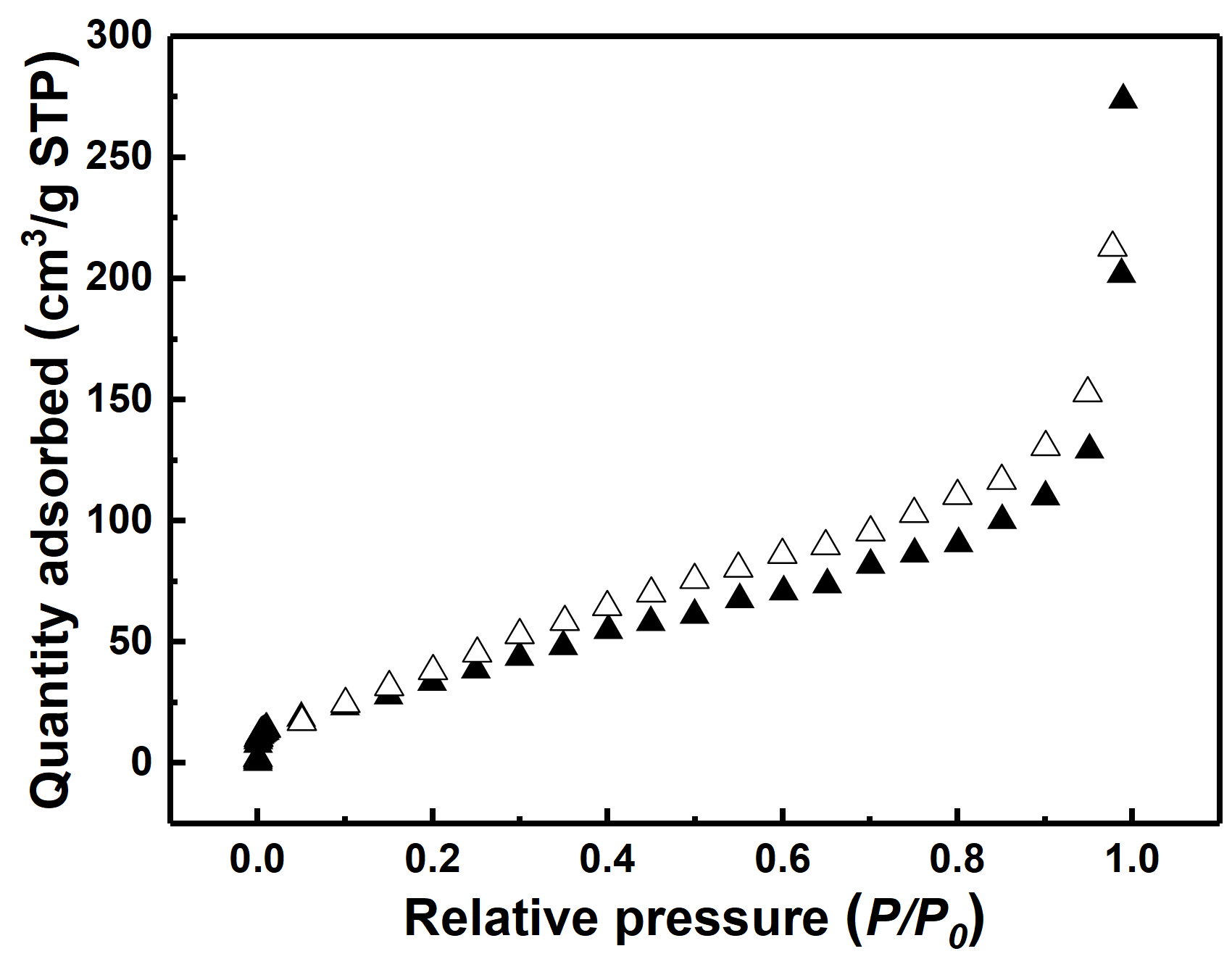


**Figure S15.** Nitrogen adsorption isotherm of the expanded graphite (EG).


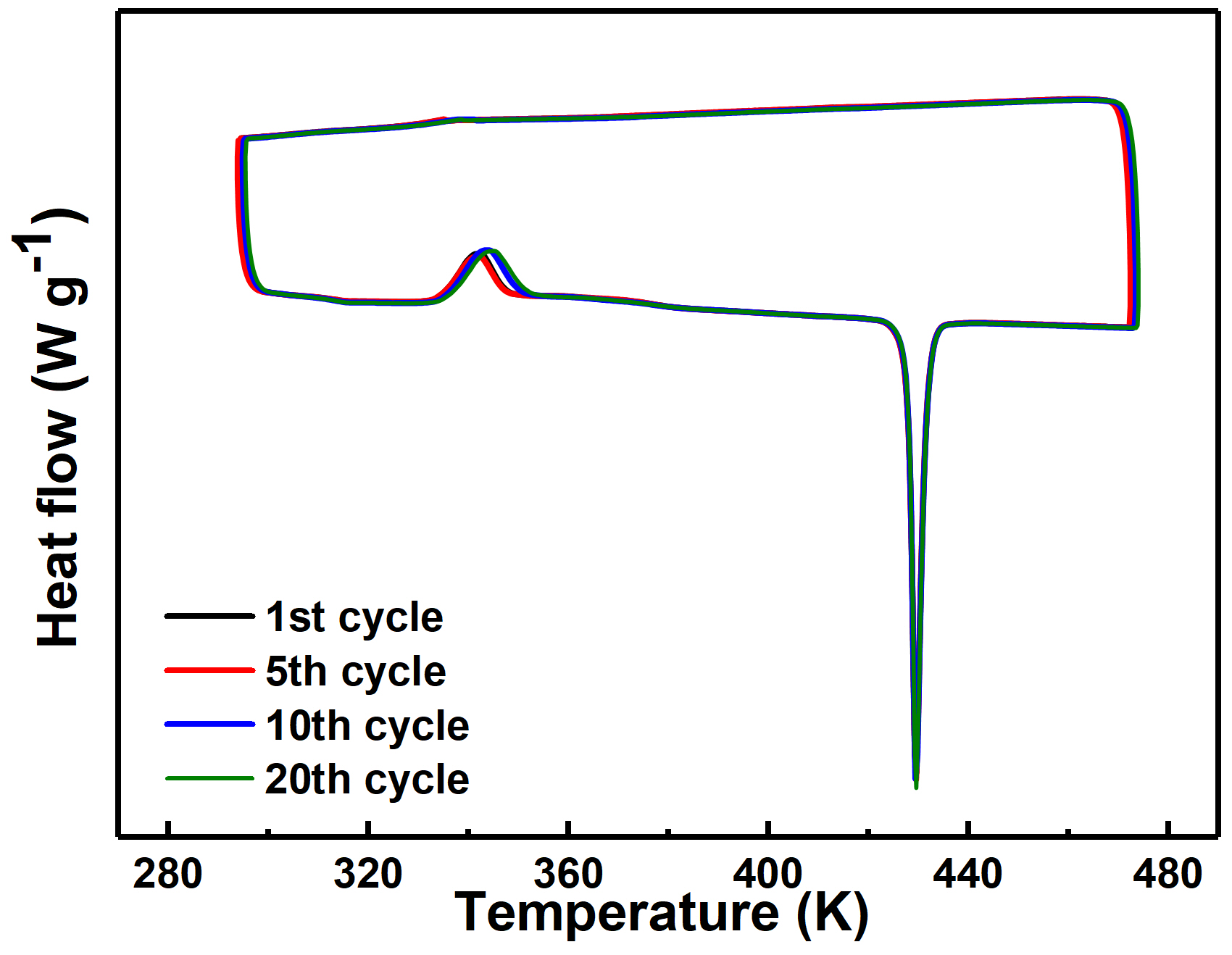


**Figure S16.** DSC curves of 1.5wt% EG/**EtP5***α* at different cycles.


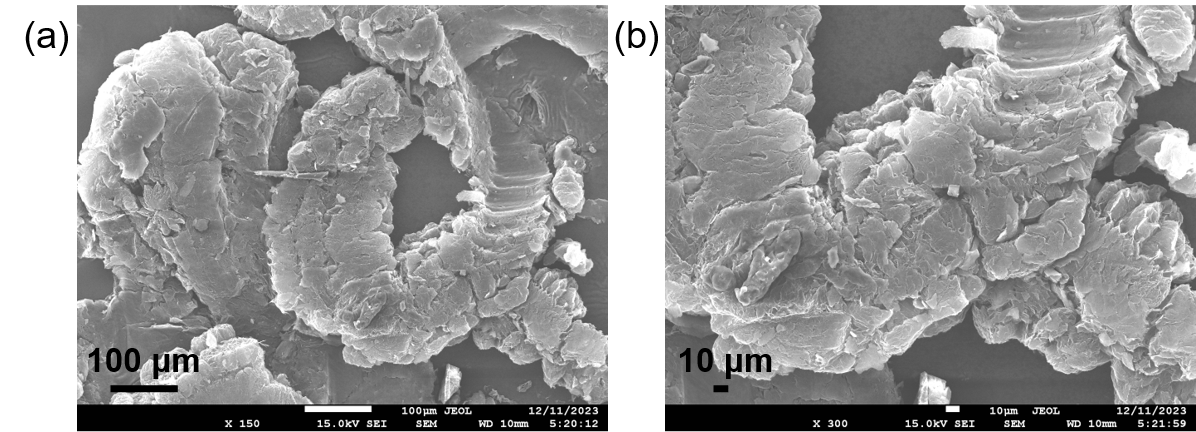


**Figure S17.** SEM images: (a) 1.5wt% EG/**EtP5***α*; (b) the enlarged view of (a).


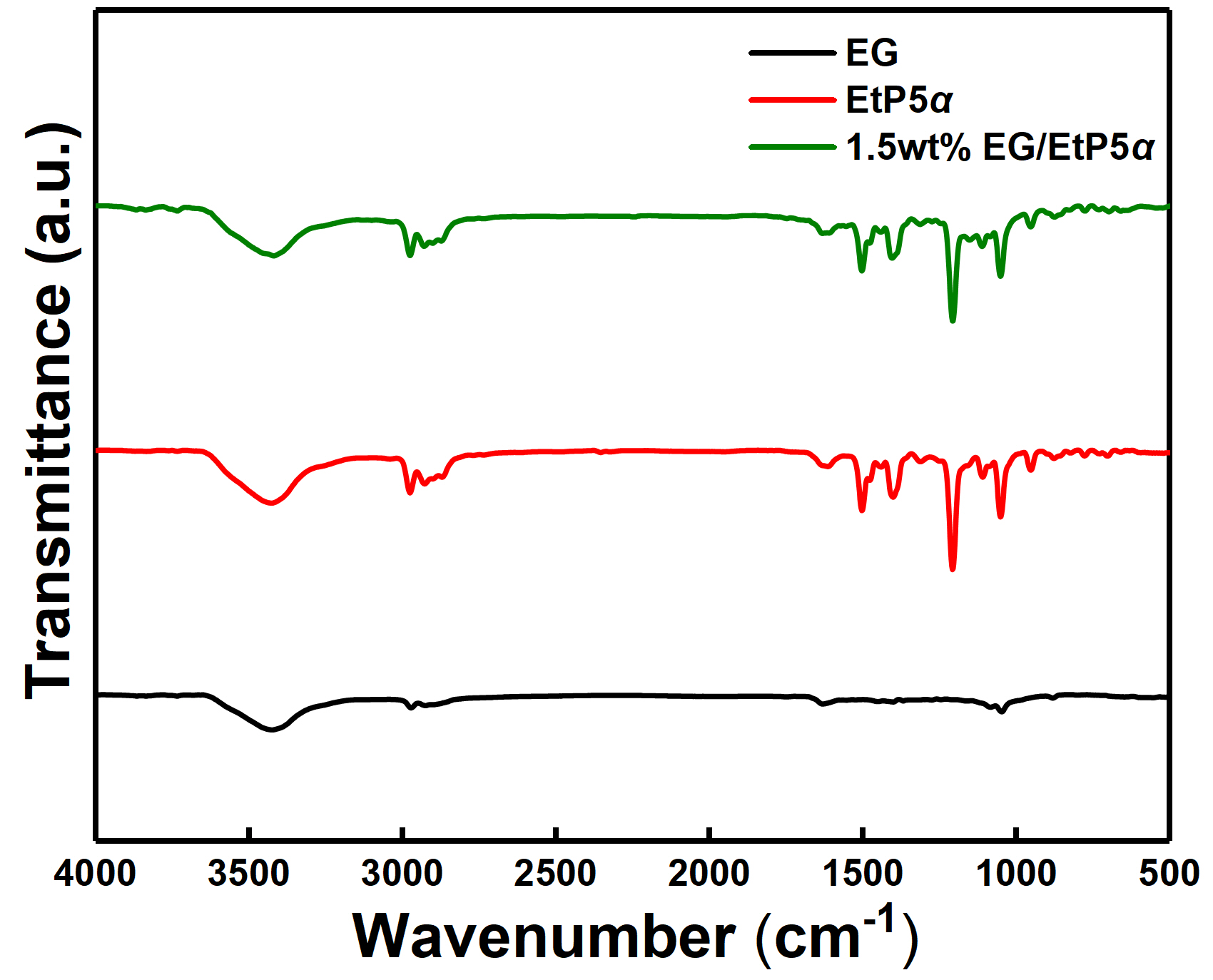


**Figure S18.** FTIR spectra of EG, **EtP5***α*, and 1.5wt% EG/**EtP5***α*.

*8. References*

1. X.-B. Hu, Z. Chen, L. Chen, L. Zhang, J.-L. Hou, Z.-T. Li, *Chem. Commun.* **2012**, *48*, 10999-11001.

2. M. Wang, J. Zhou, E. Li, Y. Zhou, Q. Li, F. Huang, *J. Am. Chem. Soc.* **2019**, *141*, 17102-17106.

3. H. K. Shin, K.-Y. Rhee, S.-J. Park, [*Compos. Part B Eng.*](https://www.x-mol.com/paper/journal/1748) **2016**, *96*, 350.

4. S. Yang, H.-Y. Shi, J. Liu, Y.-Y. Lai, Ö. Bayer, L.-W. Fan, *Nat. Commun.* **2024**, *15*, 4948.

5. G. Fang, W. Zhang, M. Yu, K. Meng, X. Tan, *Sol. Energy Mater. Sol. Cells* **2022**, *234*, 111418.

6. W. Song, S. Zhang, W. Bai, Z. Geng, X, Li, X. Ren, S. Zhang, B. Zhao, Y. Shen, *Journal of Salt Lake Research*, **2025**, *33*, 61.

7. A. Sarı, C. Alkan, Ö. Lafçı, *Sol. Energy* **2012**, *86*, 2282.

8. S. Li, L. He, H. Lu, J. Hao, D. Wang, F. Shen, C. Song, G. Liu, P. Du, Y. Wang, D. Cong, *Acta Mater.* **2023**, *249*, 118852.

9. Y. Li, Y. Kou, K. Sun, J. Chen, C. Deng, C. Fang, Q. Shi, *J. Energy Chem.* **2023**, *80*, 228.

10. J. Chen, Y. Kou, K. Sun, H. Liu, X. Zhang, C. Fang, Q. Shi, [*Mater. Today Sustain.*](https://www.x-mol.com/paper/journal/111600) **2023**, *22*, 100398.

11. B. Hess, C. Kutzner, D. van der Spoel, E. Lindahl, *J. Chem. Theory Comput.* **2008**, *4*, 435-447.

12. U. Essmann, L. Perera, M. L. Berkowitz, T. Darden, H. Lee, L. G. Pedersen, *J. Chem. Phys.* **1995**, *103*, 8577-8593.

13. B. Hess, H. Bekker, H. J. C. Berendsen, J. G. E. M. Fraaije, *J. Comput. Chem.* **1997**, *18*, 1463-1472.
